# Solubilities and Self-Diffusion Coefficients of Light *n*-Alkanes in NaCl Solutions at the Temperature Range (278.15–308.15) K and Pressure Range (1–300) bar and Thermodynamics Properties of Their Corresponding Hydrates at (150–290) K and (1–7000) bar

**DOI:** 10.1021/acs.jced.3c00225

**Published:** 2023-07-11

**Authors:** Bin Fang, Parsa Habibi, Othonas A. Moultos, Tao Lü, Fulong Ning, Thijs J. H. Vlugt

**Affiliations:** † School of Mathematics and Physics, China University of Geosciences, Wuhan 430074, China; ‡ Engineering Thermodynamics, Process & Energy Department, Faculty of Mechanical, Maritime and Materials Engineering, Delft University of Technology, Leeghwaterstraat 39, Delft 2628CB, The Netherlands; § School of Automation, China University of Geosciences, Wuhan 430074, China; ∥ Hubei Key Laboratory of Advanced Control and Intelligent Automation for Complex Systems, Wuhan 430074, China; ⊥ Faculty of Engineering, China University of Geosciences, Wuhan, Hubei 430074, China; # National Center for International Research on Deep Earth Drilling and Resource Development, China University of Geosciences, Wuhan 430074, China

## Abstract

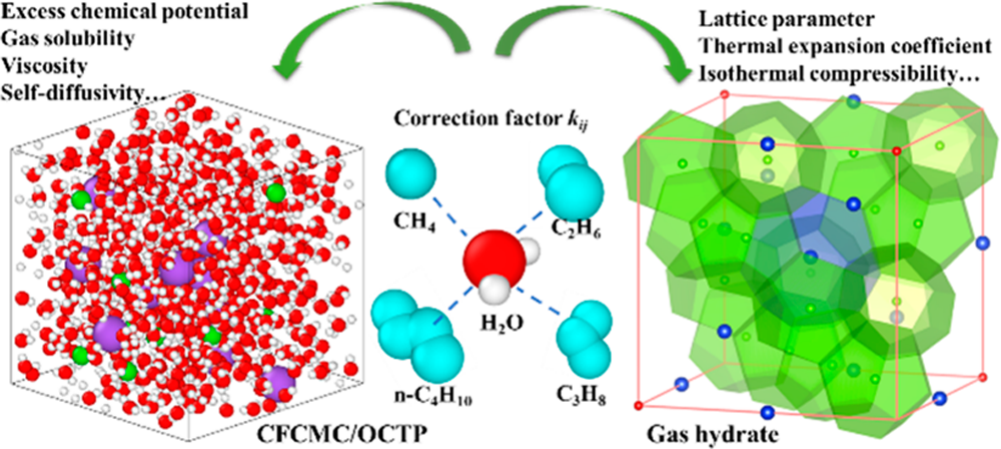

Continuous Fractional Component Monte Carlo (CFCMC) and molecular dynamics (MD) simulations are performed to calculate the solubilities and self-diffusion coefficients of four light *n*-alkanes (methane, ethane, propane, and *n*-butane) in aqueous NaCl solutions as well as the thermodynamic properties of their corresponding hydrate crystals. Correction factors *k*_*ij*_ to the Lorentz–Berthelot combining rules for alkane groups (CH_3_) and water are optimized (*k*_*ij*_ = 1.04) by fitting excess chemical potentials to experimental data at 1 bar and 298.15 K. Using these values of *k*_*ij*_, we calculate the solubilities of the four alkanes in aqueous NaCl solutions with different molalities (0–6) mol/kg at different temperatures (278.15–308.15) K and pressures (1, 100, 200, 300) bar. The diffusion coefficients of the four alkanes in NaCl solutions (0–6) mol/kg are calculated at different temperatures (278.15–308.15) K and 1 bar and corrected for the finite-size effects. The lattice parameters of the corresponding hydrates with different guest molecules are computed using MD simulations at different temperatures (150–290) K and pressures (5–700) MPa. Isothermal compressibilities at 287.15 K and thermal expansion coefficients at 14.5 MPa for the corresponding hydrates are calculated. We present an extensive collection of thermodynamic data related to gas hydrates that contribute to a fundamental understanding of natural gas hydrate science.

## 1 Introduction

Natural gas hydrates (NGHs) are ice-like crystallization compounds, in which natural gas (mainly composed of light *n*-alkanes such as methane, ethane, propane, and *n*-butane) is trapped in polyhedral cages formed by hydrogen-bonded (H-bonded) water molecules at specific pressure and temperature conditions.^1^ There are three typically identified hydrate structures on earth, and the formation of crystals mainly depends on the size of the guest molecule.^1^ Hydrates usually occur on marine sediments and permafrost regions^1^ and in pipelines.^2^ The global energy content in gas hydrates is conservatively estimated to be twice that of all other fuel sources together; thus, NGHs are considered as alternative energy resources.^3^ Moreover, hydrates have significant potential for water, energy, and environmental industrial applications, including CO_2_ capture and sequestration (CCS),^4^ hydrogen storage,^5^ seawater desalination,^6^ wastewater treatment,^7^ and gas transport.^8^ The exploitation and application of hydrates are based on accurate information about the phase change kinetics (i.e., formation and dissociation) and thermodynamic properties of NGHs that have been extensively explored. Two physical parameters that are very important for controlling the hydrate phase change are the solubilities and self-diffusion coefficients of light alkanes in water,^9^ and particularly in NaCl solutions,^10^ as hydrates often occur in seabed sediments.^1^

To date, many experimentalists have measured the solubility of methane,^11−17^ ethane,^11,17^ propane,^12,18−21^ and butane^16^ in pure water and in NaCl solutions^22−28^ at different temperatures, pressures, and molalities. Wilhelm et al.^29^ presented a review on the solubility of gases in pure water at normal pressure. In NaCl solutions, the solubility of alkanes decreases as the molality (mol salt/kg water) of the salt increases. This phenomenon is commonly referred to as the “salting out effect”.^30^ However, the data obtained from experimental measurements are still insufficient, and the majority of the studies conducted has focused on methane. For the prediction of gas solubility in solutions, equation of state (EoS) based models are often used, such as GCNLF EoS,^31^ GC EoS,^32^ and SAFT-based EoS.^33^ EoS models may not accurately describe gas solubility in complex systems and often neglect the interaction between the gas and liquid phases, resulting in potential errors in gas solubility calculations. Force field-based Monte Carlo (MC) simulations^34−36^ can effectively overcome this problem and are well-suited for estimating thermodynamic properties, such as excess chemical potentials and gas solubilities. Docherty et al.^34^ introduced a positive deviation in the energetic Lorentz–Berthelot rule to correct the interactions between methane and water and calculated the excess chemical potential of gas in water and the properties of methane hydrates. Additionally, machine learning has also been used to predict the solubilities of light alkanes in pure water and aqueous electrolyte solutions.^37^ To discuss the salting out effects of alkanes in aqueous electrolyte solutions,^38^ the Setschenow relation is often used^39^
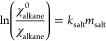
1where χ_alkane_^0^ and χ_alkane_ represent the mole fractions of alkane in pure water and electrolyte solutions, respectively, *m*_salt_ denotes the molality of salt (mol salt/kg water), and *k*_salt_ is the Setschenow salting out constant. The Setschenow constants usually decrease with temperature and depend on the nature of the gas.^40^ Within a small temperature range, the variation in the Setschenow constants with temperature can be ignored.^24^

The mass transport of light alkanes in water and an electrolyte system plays an important role in the formation and dissociation kinetics processes of hydrates, especially for the phase change rate.^41,42^ The self-diffusion coefficients of methane, ethane, propane, and *n*-butane have been measured in water^43−45^ and in aqueous electrolyte systems.^46^ Molecular dynamics (MD) simulations are widely used to calculate diffusion coefficients of pure compounds in mixtures. Pokharel et al.^47^ calculated the self-diffusion coefficients of methane, ethane, propane, and *n*-butane in water using MD simulations. Chen et al.^46^ calculated the diffusion coefficients of methane in water/brine (3.5 wt % NaCl solution). Yeh and Hummer^48,49^ discovered that the computed self-diffusion coefficients in MD simulations are strongly influenced by the system size. To correct the systematic errors, these authors developed a hydrodynamics-based finite-size correction term for classical MD simulations to adjust the self-diffusion coefficients.

Comprehending thermodynamic properties, including thermal expansion coefficients and isothermal compressibilities, is crucial for hydrate exploitation. The thermal expansion coefficient of hydrates is fundamental information for risk assessment studies concerning the mechanical stability of hydrate-bearing earth sediments.^50^ The detection of natural gas hydrates (NGHs) in sediments is typically performed using the propagation of seismic waves, which depends on the elastic properties (compressibility) of the medium.^51^ Hester et al.^52^ measured the hydrate lattice parameters for both sI and sII hydrates as a function of temperature and estimated the thermal expansion coefficient of different hydrates. Manakov et al.^53^ presented experimental data of the lattice parameters for gas hydrates as a function of pressure (0–3) GPa and obtained the gas hydrate bulk modulus. To date, many experimental results have been reported for the thermal expansion coefficient^54−60^ and compressibility^61−65^ of gas hydrates. In addition, MD simulations have proven to be a valuable tool for determining the thermodynamic properties of gas hydrates for a broad temperature and pressure range, helping to explain the variations in thermodynamic properties between hydrate systems containing different guest molecules.^66,67^

Several experimental and simulation studies have been conducted for obtaining the solubilities and transport properties of alkanes in pure water and aqueous NaCl solutions. These data are for limited temperature/pressure ranges; such data at hydrate formation conditions, and particularly for NaCl solutions data, are largely lacking. The remainder of this study is structured as follows. In Section 2, we first show the force field, explain how to calculate solubilities and self-diffusion coefficients, and build hydrate crystals with different guest molecules to calculate thermodynamics properties using classical MC and MD simulations. In Section 3, we introduce a correction factor *k*_*ij*_ between alkane groups (CH_3_) and water by calculating the excess chemical potential at 298.15 K and 1 bar. Using this correction factor, we calculated the solubilities and diffusion coefficients of the four alkanes in NaCl solutions at temperatures ranging from 278.15 to 308 K, pressures ranging from 1 to 300 bar, and molalities ranging from 0 to 6 mol/kg. We also calculated the compressibility and expansion coefficients for the four corresponding hydrates. Finally, Section 4 summarizes the conclusions drawn from the study.

## 2 Methods

### 2.1 Force Fields

Water is modeled using the TIP4P/2005 force field.^68^ This model predicts the densities, viscosities, and self-diffusion coefficients of water with high accuracy over a wide temperature range and performs well in an aqueous salt solution system containing gases.^34,36^ The description of the CH_4_ molecule and its interaction with water are handled in the same way as in Docherty’s study.^34^ The TraPPE-UA force field^69^ is used for the other three light alkane molecules i.e., C_2_H_6_, C_3_H_8_, and n-C_4_H_10_. The Madrid-2019 force field proposed by Zeron et al.^70^ is used for Na^+^ and Cl^–^ with scaled changes, which considers the polarization effect and improves the description of the salt system in water. All force field details are provided in Table S1, Table S2, and Table S3 in the Supporting Information. The chemical formulas, CAS numbers, and force fields of all species used in the simulations are listed in Table 1. The nonbonded intermolecular interactions are handled by Lennard-Jones (LJ) and Coulombic potentials, which are expressed as follows^71^
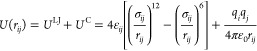
2where *r*_*ij*_ is the distance between particles *i* and *j*, *ε*_*ij*_ is the depth of the LJ potential well, and *σ*_*ij*_ is the distance at which the pair potential energy *U* is zero. An unshifted potential (+tail corrections) was used so that the force field used in both the MC and MD simulations was identical. The energy (*ε*_*ij*_) and distance (*σ*_*ij*_) parameters for the cross interaction between the groups in the alkanes and water were described by the modified version of the Lorentz–Berthelot combining rules (a correction factor *k*_*ij*_ is applied to adjust the energetic cross interactions (*ε*_*ij*_)):^72^
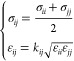
3

**Table 1:** Description of All the Species Used in the Simulations

Chemical name	Chemical formula	CAS number	Force field
Water	H_2_O	7732-18-5	TIP4P/2005^68^
Methane	CH_4_	74-82-8	Hirschfelder^34^
Ethane	C_2_H_6_	74-84-0	TraPPE^69^
Propane	C_3_H_8_	74-98-6	TraPPE^69^
Butane	C_4_H_10_	106-97-8	TraPPE^69^
Sodium ion	Na^+^	7440-23-5	Madrid-2019^70^
Chlorine ion	Cl^–^	16887-00-6	Madrid-2019^70^

Deviations from this rule can be accounted for by using values of *k*_*ij*_ that differ from 1. For the energetic cross interactions between alkane groups and ions, as well as between water and ions, the value of *k*_*ij*_ in eq 3 is set to 1, which corresponds to the conventional Lorentz–Berthelot combining rule.

### 2.2 Monte Carlo Simulations

The Continuous Fractional Component Monte Carlo (CFCMC) technique in the NPT ensemble is used to calculate the excess chemical potentials and solubilities of the four light alkanes in aqueous NaCl solutions. All MC simulations are performed by the open-source Brick-CFCMC software.^73−75^ A so-called fractional molecule of each component type was introduced into the simulation. All of the MC simulation systems contained both water and ion molecules. The total number of water molecules in each system is fixed at 270, while the number of ions is determined by the NaCl molality present in the solution. An expanded conventional NPT ensemble was used to control the pressure and temperature. The interaction potential for the fractional molecule is determined by the fractional parameter λ∈[0,1]. When λ is equal to zero, no interactions between the fractional molecules and surrounding molecules are present (i.e., the fractional molecule acts as an ideal gas molecule). When λ is equal to one, this fractional molecule acts as a “whole molecule” (i.e., full interaction between the fractional molecule and surrounding molecules). The excess chemical potential of alkanes in aqueous NaCl solutions can be calculated from the probability distribution of λ^76^
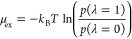
4where μ_ex_ is the excess chemical potential with respect to ideal gas, *T* is the temperature, and *p*(λ = 0) and *p*(λ = 1) are the probabilities when λ equals 0 and 1, respectively.

The Henry coefficient *K*_*H*_ can be computed from^39^
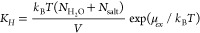
5where *N*_H_2_O_ and *N*_salt_ are the molecular number of water and salt, respectively, and *V* is the volume of the system. At low pressure, the concentration of the solute in a dilute solution is directly proportional to its mole fraction (*x*). This relationship is described by Henry’s law, which can be expressed as follows:
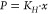
6

At high pressures, the solubility of light alkanes is computed by using the Gibbs Ensemble, where a fractional molecule is used for the insertion and deletion of light alkane molecules. The fugacity coefficients for the light alkanes at different temperatures and pressure conditions are computed using REFPROP software.^77^Table S4 in the Supporting Information contains a list of all fugacity coefficients.

During MC simulations, both Lennard-Jones and Coulombic interactions were cut off at 9 Å, and analytical tail corrections are implemented. The Ewald method was used to calculate the long-range electrostatic interaction energy.^78^ The probabilities of selecting trial moves were as follows: 34% translations, 24% rotations, and 10% changes in the geometry of molecules (angle and dihedral), 20% changes in the fractional parameter, and 2% volume changes, 1 × 10^4^ cycles to initialize the system, 5 × 10^5^ cycles were performed for system equilibration, and 1 × 10^6^ cycles were performed for production in the simulations. A cycle is defined as a set of N trial moves. For excess chemical potential calculations, the simulation temperature range is 278.15–308.15 K, and the pressure is 1 bar. For solubility calculations, the simulation temperatures range is 278.15–308.15 K, and the pressure is 1–300 bar.

### 2.3 Molecular Dynamics Simulations

**Figure 1:**
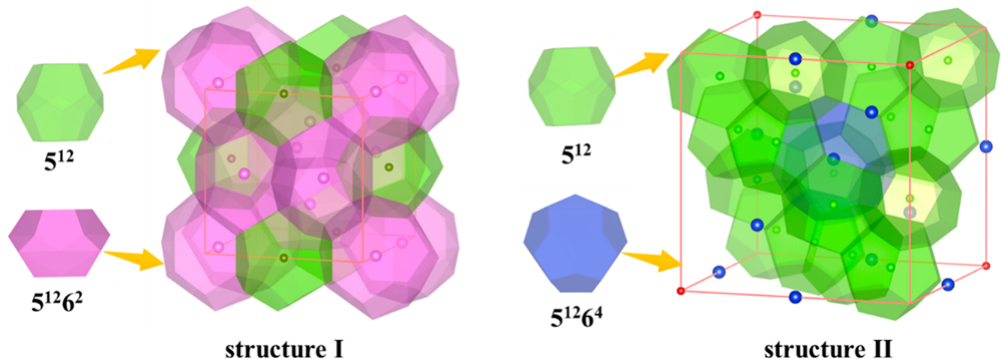
Host structures of sI and sII unit hydrate cells: 5^12^ cages colored in green, 5^12^6^2^ cages colored in purple, and 5^12^6^4^ cages presented in blue.

MD simulations are carried out using the large-scale atomic/molecular massively parallel simulator (LAMMPS) to calculate the transport properties of light *n*-alkanes in aqueous NaCl solutions and the thermodynamic properties of their corresponding gas hydrates. To simulate the transport properties, the initial MD systems consist of *n*-alkane, water, and ion molecules. In each system, there is only one *n*-alkane molecule and 555 water molecules, and the number of ions is determined by NaCl molality. The initial step in simulating the thermodynamic properties involves creating a molecular-level crystal structure of the corresponding alkane hydrate. Based on experimental results, methane and ethane hydrates are often present in the sI structure, whereas propane and *n*-butane hydrates are in the sII structure. It should be noted that the formation of *n*-butane hydrates requires a “help gas” (e.g., CH_4_);^79,80^ therefore, we calculated the thermodynamic properties of a butane+methane binary hydrate. The coordinates of the host water molecules in both crystal structures are determined by Takeuchi’s work,^81^ in which the oxygen atom positions of the hydrate are determined by XRD results,^82^ and the hydrogen orientation in water molecules is adjusted to adhere to the Bernal-Fowler rule and minimize both the structural potential energy and dipole moment. Host structures of sI and sII unit hydrate cells are shown in Figure 1. Methane and ethane molecules fully occupy the 5^12^ and 5^12^6^2^ cages in the sI structure of hydrate. Propane and butane molecules are located in the center of the large 5^12^6^4^ cage, and the “help gas” CH_4_ occupies the 5^12^ cages in the sII structure of the hydrate. The initial MD configurations of methane and ethane hydrates are in a 3 × 3 × 3 supercell with a 12.03 Å lattice parameter for an sI unit cell.^81^ The propane and butane+methane hydrates’ initial configurations are in a 2 × 2 × 2 supercell, with a 17.31 Å lattice parameter for an sII unit cell.^81^ For this binary hydrate, methane molecules are occupied in the sixteen 5^12^ cages, and butane molecules are put in the eight 5^12^6^4^ cages in the sII unit hydrate cell.

For all systems, the cutoff radius for both the LJ and the short-range part of the Coulombic interactions is set at 12 Å. The particle–particle particle-mesh (PPPM)^83^ method is used to calculate the long-range electrostatic interactions, with a relative error of 10^–5^. The initial configurations, for both sI and sII structures, are first subjected to minimization using the Polak-Ribiere version of the conjugate gradient (CG) algorithm.^84^ An equilibration simulation of 2 ns is followed by an isothermal–isobaric (constant-temperature, constant-pressure) ensemble simulation. The Nose-Hoover thermostat and barostat are used for temperature and pressure coupling with thermostat and barostat constants of 0.1 and 1.0 ps, respectively.^85^ The Verlet algorithm^86^ was utilized to integrate Newton’s equations of motion with a time step of 1 fs. The periodic boundary conditions were applied in all directions of the systems.

Transport properties were calculated by on-the-fly computation using the transport property plugin (OCTP) in LAMMPS.^87^ The plugin calculates the transport coefficients based on the mean-squared displacements (MSDs) of the dynamical properties obtained from the MD simulation. Specifically, the transport coefficients are determined as the slopes of the MSDs, which are plotted as linear functions of time. The self-diffusivity *D*_*Self*_^*MD*^ and viscosity η can be directly computed in MD simulations using Einstein relations^88^
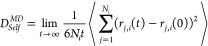
7and
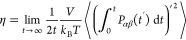
8where *N*_*i*_ is the number of the specific molecule *i* in the system, and *r*_*j*,*i*_(*t*) and *r*_*j*,*i*_(0) are the positions of the *j-*th molecule of species *i* at time *t* and 0, respectively. *P*_*αβ*_(*t*′) denotes the off-diagonal elements of the stress tensor at time *t*; *V* is the system volume, and the brackets ⟨...⟩ are the ensemble averages.

To remove the effect of the system size in MD simulations, Yeh and Hummer^48^ deduced a finite-size correction term using hydrodynamics, defined as the Yeh-Hummer (YH) correction
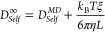
9where *D*_*Self*_^*MD*^ represents the finite self-diffusion coefficient obtained from MD simulations, *k*_B_ is the Boltzmann constant, *T* is the absolute temperature, η is the shear viscosity obtained from the simulation (the value of η is independent of the system size),^48,89−91^ and ξ is a constant with a value of 2.837298 for periodic lattices, as discussed by previous studies.^49,92^

For transport property calculations, the simulation temperature range is 278.15–308.15 K, and the pressure is 1 bar. For the simulations of expansion coefficients for the four corresponding hydrates, the temperature range is 150–300 K at 145 bar. For the calculation of gas hydrate compressibility, the temperature is fixed at 287.15 K, and the pressure range is 50–7000 bar.

## 3 Results and Discussion

### 3.1 Correction Factor *k*_*ij*_ for Alkane Groups and Water

In Docherty’s work,^34^ the correction factor (*k*_*ij*_ = 1.07) for methane and water was optimized from the simulation of the excess chemical potential of methane in water using Widom’s test particle method. Using this factor, we utilized a CFCMC simulation to compute the excess chemical potentials of methane in water at 1 bar across a range of temperatures. The results are shown in Figure S1 of the Supporting Information. Compared to Docherty’s simulation^34^ and Paschek’s experimental^93^ results, the numerical results of excess chemical potentials of methane in water are accurate with a small error at 298.15 K and 1 bar. Therefore, we chose this value of *k*_*ij*_ to describe the methane-water interaction and calculate the excess chemical potentials, solubilities, and self-diffusion coefficients of methane in NaCl solutions at different molalities, temperatures, and pressures.

**Figure 2:**
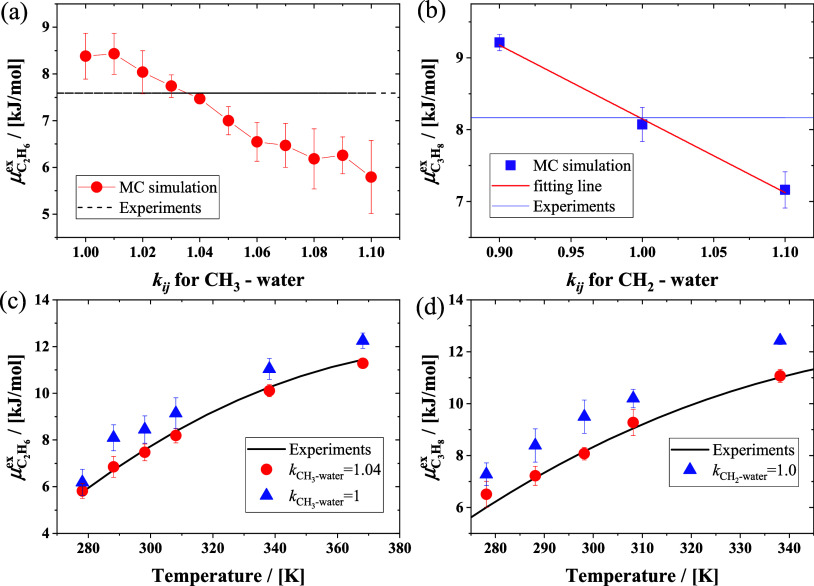
Comparison of simulated and experimental excess chemical potentials of ethane (a) and propane (b) in water by changing the LJ *k*_*ij*_ parameter of the CH_3_ (a) and CH_2_ (b) groups and water molecules at 298.15 K and 1 bar. The excess chemical potential of ethane (c) and propane (d) in water as a function of temperature at 1 bar by MC simulation using different scaling *k*_*ij*_ parameters and the experimental results.^29^

The simulation results for the excess chemical potential of ethane in water using different force field combinations for ethane and water molecules at 298.15 K and 1 bar are shown in Table S5 of the Supporting Information. The reference value of the excess chemical potential was calculated using the experimental ethane solubility^29^ which is equal to 7.59 kJ/mol. As shown in Figure 2c, the difference between the simulation value and the reference results (calculated using the solubility experimental value) appears systematically (all the calculated values of the excess chemical potential are larger than the reference values at different temperatures) with temperature. This phenomenon is similar to the calculation of the methane excess chemical potential in water.^34^ Therefore, we increased the well depth of the CH_3_ group–water LJ interaction to correct the interactions between ethane and water, which is the same as in the study by Docherty.^34^ As shown in Figure 2a, by increasing the value of *k*_*ij*_, the value of the excess chemical potential of ethane () in water at 298.15 K and 1 bar decreases. The computed value of  is the closest to the experimental fitting value when *k*_*ij*_ = 1.04. The difference between the simulation and reference value (calculated using the solubility experimental value) is only 0.12 kJ/mol (relative error 1.58%). Using this *k*_*ij*_, the excess chemical potentials of ethane in water at 1 bar and different temperatures are computed. The simulation results are listed in Figure 2c. Clearly, the combination of the TIP4P/2005 model for water, the TraPPE model for ethane, and the modification of the interaction energy of the CH_3_ group and water oxygen in the Lorentz-Bethelot combining rules yields good agreement with the reference values of the excess chemical potential for the whole temperature range.

The excess chemical potentials of propane in water at different temperatures are shown in Figure 2d. Without optimizing the interaction between propane and water, the simulation results for μ_C_3_H_8__^ex^ are larger than those of the experimental value. This indicates that the polarization of propane in water should not be ignored in excess chemical potential calculations. In the propane-water system, both CH_3_ group-water and CH_2_ group-water LJ interactions should be considered. Here, *k*_*ij*_ for the interaction between CH_3_ and water is fixed at 1.04; similarly, we vary the value of *k*_*ij*_ between CH_2_ and water, and the results are shown in Figure 2b. The performance is the best when *k*_*ij*_ for the interaction between the CH_2_ group and water is equal to 1. This indicates that the interaction between propane and water is mainly determined by the two CH_3_ groups. Using the two *k*_*ij*_ values, the simulated values at different temperatures and 1 bar are in agreement with the experimental results. Therefore, to compute the excess chemical potentials and solubilities of the light *n*-alkanes in NaCl solutions, we used the correction factor *k*_*ij*_ = 1.04 to correct the interaction between the CH_3_ group and water and *k*_*ij*_ = 1 for the interaction between the CH_2_ group and water at different temperatures and pressures.

### 3.2 Excess Chemical Potentials and Solubilities of the Four Alkanes in NaCl Aqueous Solutions

**Figure 3:**
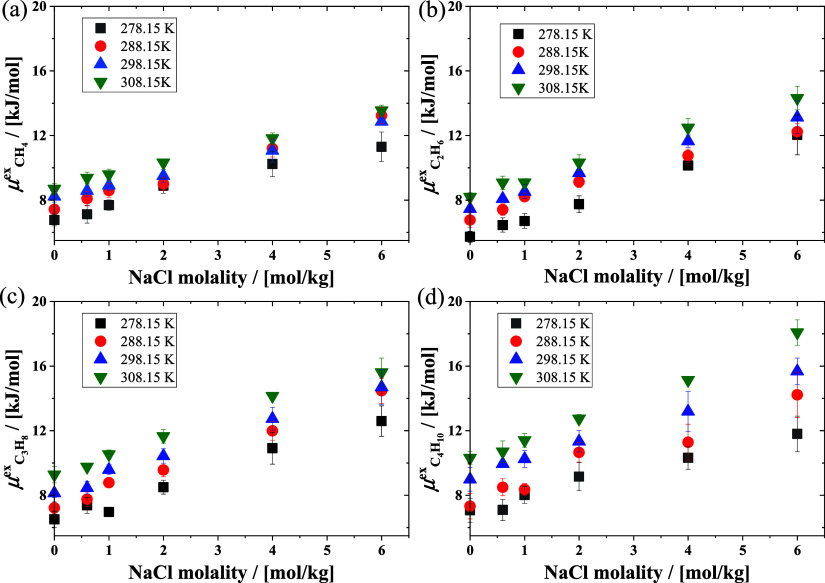
Excess chemical potentials of (a) methane, (b) ethane, (c) propane, and (d) butane in NaCl solutions as a function of the temperature and NaCl molality.

In Figure 3, the computed excess chemical potentials of the four *n*-alkanes in the NaCl solution as a function of NaCl molality at 1 bar and different temperatures are shown. For a fixed molality, the higher the temperature, the larger the excess chemical potential of alkanes in NaCl solutions. By increasing the NaCl molality, the excess chemical potential of alkanes in the NaCl solution increases linearly. At identical conditions of temperature and molality, the excess chemical potentials of the four alkanes exhibit variations, especially at high concentrations of NaCl in water.

**Figure 4:**
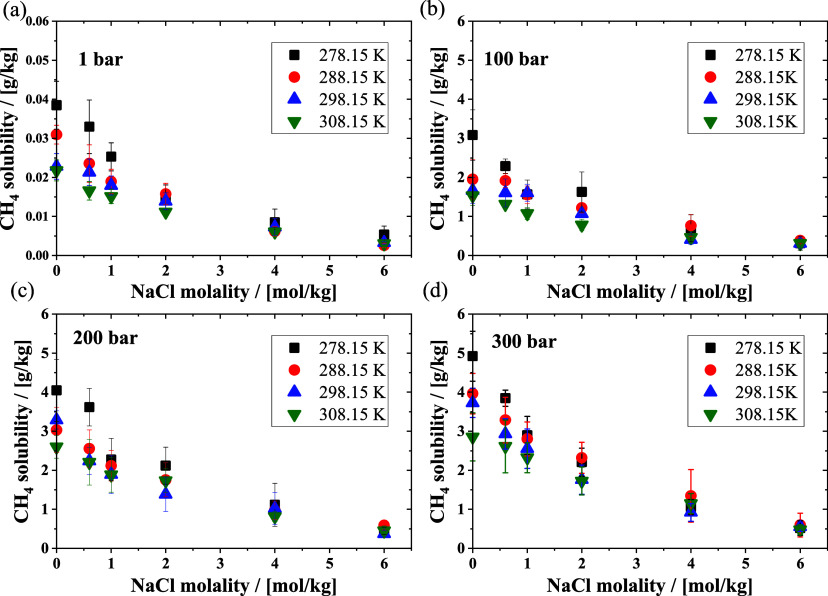
Solubilities of methane in NaCl solutions as a function of NaCl molality in solution at different pressures (a) 1, (b) 100, (c) 200, and (d) 300 bar and temperatures in the range (278.15–308.15) K.

Based on the excess chemical potentials shown in Figure 3, we can obtain the Henry coefficients using Eq 5 and the solubilities of the four alkanes in the NaCl solution by Eq 6 at 1 bar. The solubilities of methane in the NaCl solution at 1 bar are shown in Figure 4a. Methane solubilities in the NaCl solution as a function of temperature and NaCl molality at 100, 200, and 300 bar are shown in Figures 4b, 4c, and 4d, respectively. As expected, the solubility of methane decreased with decreasing pressure and increasing temperature. As shown in Figures 4a and 4b, the solubility of methane increases significantly (ca. 2 orders of magnitude) from 1 to 100 bar under the same temperature conditions. At even higher pressures (Figures 4b, 4c, and 4c), increasing the pressure from 100 to 300 bar leads to slightly higher alkane solubilities. Besides the temperature and pressure trends, the experimentally observed salting out phenomena are captured by the simulations.^30^ When the molality of NaCl in water increases, the solubility of methane decreases. At low NaCl molalities (below 2 mol/kg), increasing the temperature from 278.15 to 308.15 K leads to lower methane solubilities at the same pressures, so the temperature effect cannot be ignored. As the molality of a solution increases, the solubility becomes less affected by changes in the temperature and more influenced by the concentration of the salts. The effect of pressure on methane solubility in water also weakens with increasing NaCl molalities.

**Figure 5:**
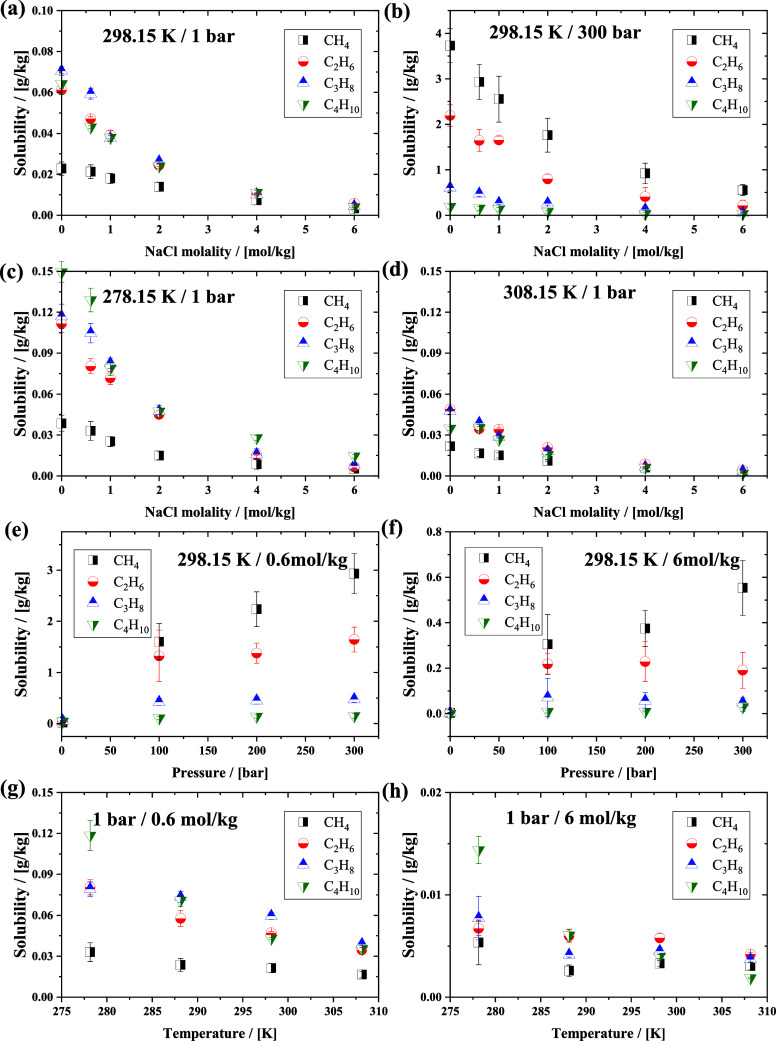
Solubilities of the four light *n*-alkanes as a function of NaCl molality at (a) 298.15 K and 1 bar, (b) 298.15 K and 300 bar, (c) 278.15 K and 1 bar, (d) 308.15 K and 1 bar; Solubilities of the four light *n*-alkanes as a function of pressure at (e) 298.15 K and 0.6 mol/kg NaCl molality, (f) 298.15 K and 6 mol/kg NaCl molality; Solubilities of the four light *n*-alkanes as a function of temperature at (g) 1 bar and 0.6 mol/kg NaCl molality, (h) 1 bar and 6 mol/kg NaCl molality.

The solubilities of ethane, propane, and butane *n*-alkanes as a function of NaCl molality at different temperatures and pressures are shown in Figures S2–S4 of the Supporting Information. Figure 5 shows the solubilities of the four alkanes at various temperatures, pressures, and NaCl molality in the solution. As shown in Figures 5a-5d, the solubilities of alkanes decrease as the molality of NaCl in water increases. Additionally, Figures 5e and 5f show that decreasing pressure also decreases solubility, while Figures 5g and 5h show that solubility decreases as the temperature increases. The simulation results clearly indicate the salting out effect for all alkanes in the NaCl solutions. At a temperature of 298.15 K and a pressure of 1 bar (Figure 5a), methane exhibits the lowest solubility among the four alkanes, and the solubilities of the other three alkanes are comparable to each other at the same molalities. At 300 bar (Figure 5b), the differences among the solubilities of the four types of alkanes are very pronounced; the solubilities decrease with the carbon number. When the pressure is increased from 1 to 300 bar, the solubility of alkanes increases significantly (1–2 orders of magnitude). As can be seen in Figures 5c and 5d, the solubilities of the four alkanes in the NaCl solution decrease as the temperature increases. At higher NaCl molalities, the difference in the solubility of the four alkane molecules in water becomes less pronounced. At ca. 6 mol/kg, which is close to the saturation solubility of NaCl in water, the solubilities of the four alkanes become almost the same. At 298.15 K, the solubility of the four alkanes increases significantly if the pressure increases from 1 to 100 bar (Figures 5e and 5f). The solubility of methane in the NaCl solution increases further in the pressure range of 100–300 bar, whereas for the other three alkanes, the solubilities vary only weakly. This indicates that the solubility of *n*-alkanes in the NaCl solution is sensitive to pressure within a specific range, which depends on the alkane. This result holds for both the low and the high NaCl molalities. As shown in Figures 5g and 5h, at 1 bar and low NaCl molalities (0.6 mol/kg), the differences between the solubilities of the four types of alkanes are pronounced at the same temperatures; at high NaCl molalities (6 mol/kg), the differences in solubilities decrease, and the solubilities become mainly dependent on the NaCl molality for all four types of alkanes.

**Figure 6:**
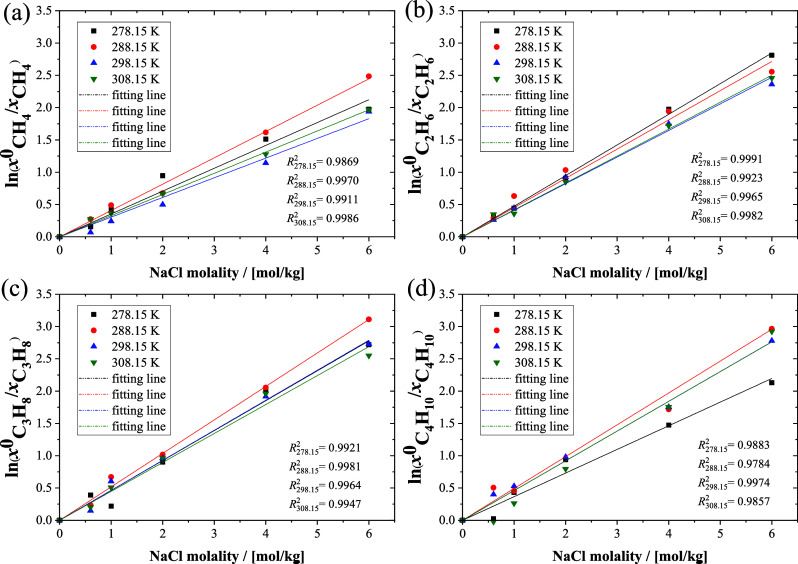
Ratio of logarithms of molar fractions of methane computed by MC simulations for the four alkanes as a function of NaCl molality in water as well as the fitting lines.

By calculation of the solubilities of alkanes in the NaCl solution at different molalities, the empirical Setschenow equation (Eq 1) can be used to describe the salting out effect. The ratios of logarithms of the mole fractions of the alkanes as a function of NaCl molalities computed in the CFCMC simulations are shown in Figure 6. We used a linear function to establish the relationship between the ratio and molality, and the fitting lines, which have a high determination coefficient, are shown in Figure 6. In Table 2, the Setschenow coefficients of the four alkanes are listed at different temperatures and 1 bar. The simulation results are in good agreement with the reference results.^94^ As expected, the salting out coefficient is almost temperature independent for the four light alkanes.

**Table 2:** Setschenow Coefficients *k*_salt_ of Alkanes as a Function of Temperature at 1 bar

	Methane	Ethane	Propane	Butane
278.15 K	0.35 ± 0.018	0.47 ± 0.006	0.463 ± 0.018	0.37 ± 0.017
288.15 K	0.40 ± 0.010	0.45 ± 0.017	0.51 ± 0.01	0.493 ± 0.03
298.15 K	0.305 ± 0.013	0.412 ± 0.01	0.465 ± 0.012	0.461 ± 0.01
308.15	0.327 ± 0.005	0.416 ± 0.007	0.448 ± 0.014	0.46 ± 0.02
ref value^94^	0.319	0.399	0.461	0.521

### 3.3 Self-Diffusion Coefficients of the Four Alkanes in NaCl Aqueous Solutions

**Figure 7:**
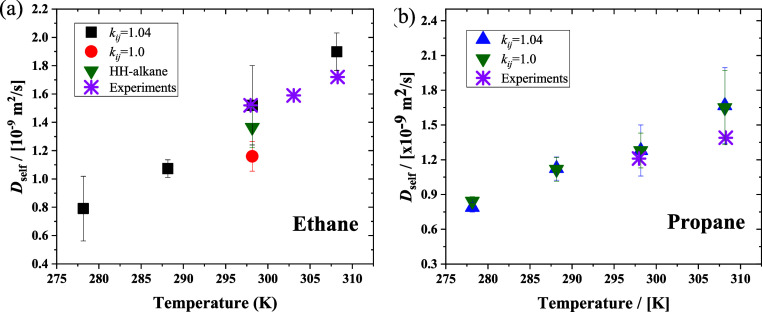
Self-diffusion coefficients of (a) ethane and (b) propane in water as a function of the temperature at 1 bar. The diffusivities are corrected by the finite-size effects.

In Section 3.1, we showed the energy interaction between the CH_3_/CH_2_ group and water by CFCMC calculation of the excess chemical potential at 298.15 K and 1 bar. The correction factor *k*_*ij*_ for CH_3_ and water is 1.04, and the correction factor *k*_*ij*_ for CH_2_ and water is 1. Here, MD simulations were performed to calculate the self-diffusion coefficients of alkanes in pure water at different temperatures using eq 9. The results for ethane and propane with and without using the correction factor *k*_*ij*_ are shown in Figure 7, along with the experimental results.^43−45^ For ethane (Figure 7a), the computed self-diffusion coefficients using the optimized value *k*_*ij*_ = 1.04 in the Lorentz-Bethelot combining rules are in agreement with the experiments at 298.15 K and 1 bar. The performance of the force field combination (TIP4P/2005 for water and TraPPE for ethane) with a correction factor is better than that of the HH-alkane force field and TraPPE without modification (*k*_*ij*_ = 1 for CH_3_ and water). At 308.15 K, the difference in the self-diffusion coefficient between the simulation and experimental results becomes pronounced, which may be attributed to the correction factor being optimized at 298.15 K and 1 bar, at different temperatures; this value of *k*_*ij*_ may not describe the interaction between ethane and water well. The self-diffusion coefficients for propane are listed in Figure 7(b). Practically, there is no obvious difference in the self-diffusion coefficients when using the correction factor *k*_*ij*_. These results indicate that the correction factor fitting by the excess chemical potential also performs well in the calculation of diffusion coefficients.

**Figure 8:**
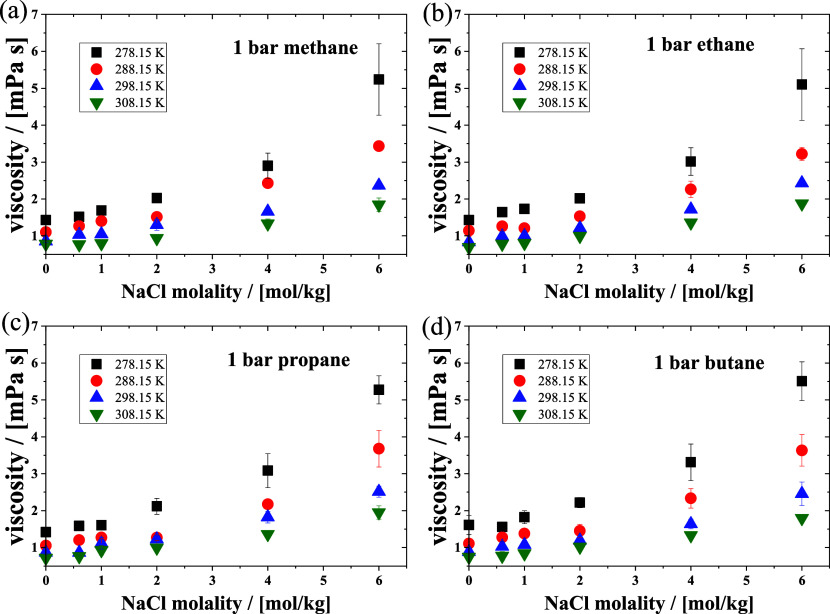
Simulated viscosities of NaCl solutions with (a) methane, (b) ethane, (c) propane, and (d) butane at different temperatures as a function of NaCl molality.

Using the correction factor *k*_*ij*_ obtained in this work, the calculated viscosities for the four systems as a function of NaCl molality in water at 1 bar and various temperatures are shown in Figure 8. The shear viscosity of the system increases with increasing NaCl molality and temperature. The contribution of alkanes to the shear viscosity of the system is relatively small because alkanes are nonpolar molecules that do not engage in hydrogen bonding or ionic interactions with water molecules or ions. Since viscosity is the result of intermolecular interactions in solution, and there is only a single alkane molecule in each MD simulation, the effect of alkane-alkane on the viscosity of a NaCl aqueous solution is absent.

**Figure 9:**
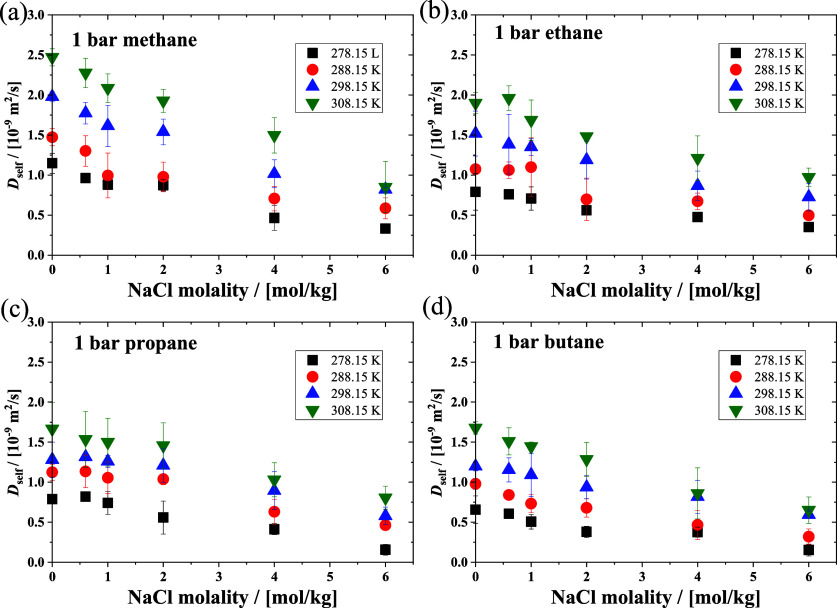
Computed self-diffusion coefficients of (a) methane, (b) ethane, (c) propane, and (d) butane in NaCl aqueous solutions as a function of NaCl molality at different temperatures and 1 bar.

The self-diffusion coefficients of the four alkanes in the NaCl aqueous solution as a function of NaCl molalities at different temperatures are shown in Figure 9. With an increase in temperature, the self-diffusion coefficients of the alkanes increase, and the self-diffusion coefficients decrease with increasing NaCl molality. At the same temperature and NaCl molality, the self-coefficient decreases with an increase in the number of carbons in the *n*-alkane.

### 3.4 Thermodynamics Properties of the Alkanes’ Corresponding Hydrate Crystals

**Figure 10:**
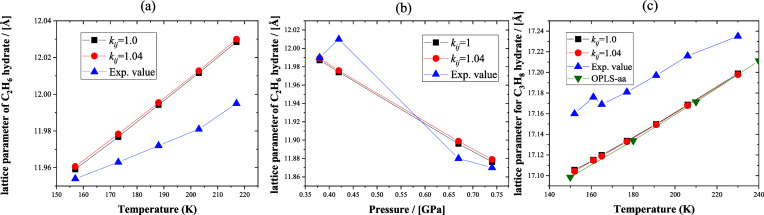
Lattice parameters of (a,b) ethane and (c) propane hydrates at various temperatures and pressures by using MD simulations, along with the experimental measurement results.

Gas hydrates are composed of *n*-alkanes and water in the solid phase. We calculated the lattice parameter of ethane and propane hydrates at different temperature and pressure conditions as shown in Figure 10. The results are compared with the experimental measurements of Hester^52^ and Manakov.^53^ The simulation results are consistent with the experimental results for a wide temperature and pressure range. The simulation results obtained using *k*_*ij*_ = 1.0 (without correction) and *k*_*ij*_ = 1.04 for the CH_3_ group and water interaction are almost identical. This indicates that the correction factor optimized in this study also describes the hydrate crystal system.

**Figure 11:**
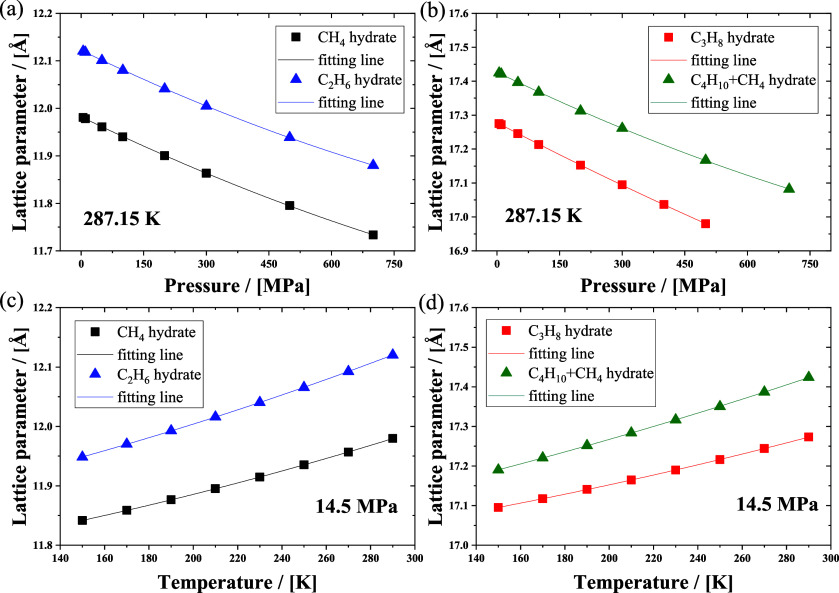
Lattice parameters of the sI and sII structure hydrates with the four *n*-alkanes as a guest molecule as a function of temperature and pressure. Methane and ethane hydrates are in structure I and shown in parts (a) and (c); propane and *n*-butane+methane hydrates are in structure II, and the simulation results are shown in parts (b) and (d).

To investigate the effect of pressure and temperature on the lattice parameter, the temperature was first set to 287.15 K, and the pressure was varied from 5 to 700 MPa. The lattice parameters of the methane hydrate, ethane hydrate, propane hydrate, and butane+methane binary hydrate are shown in Figures 11(a) and 11(b). The lattice parameters of the hydrate decrease with an increasing pressure. The H-bonded network framework formed by host water molecules in hydrate crystals can be compressed, while the guest–host interactions ensure the stability of the crystal at high pressure. Subsequently, the pressure was set to 14.5 MPa, and the temperature was varied from 150 to 290 K. Lattice parameters of those hydrates are shown in Figures 11(c) and 11(d). The lattice parameters of the hydrate increase with an increasing temperature. Both methane and ethane hydrates form structure I. The lattice parameter of ethane hydrate is larger than that of methane hydrate at the same temperature and pressure. Propane hydrate and butane+methane binary hydrate form structure II. The lattice parameter of butane+methane binary hydrate is larger than that of propane hydrate at the same temperature and pressure. The difference in the lattice parameter of hydrate is induced by the guest–host interactions. A guest molecule with a large van der Waals volume may cause the crystal to expand.^66,67,95^

**Figure 12:**
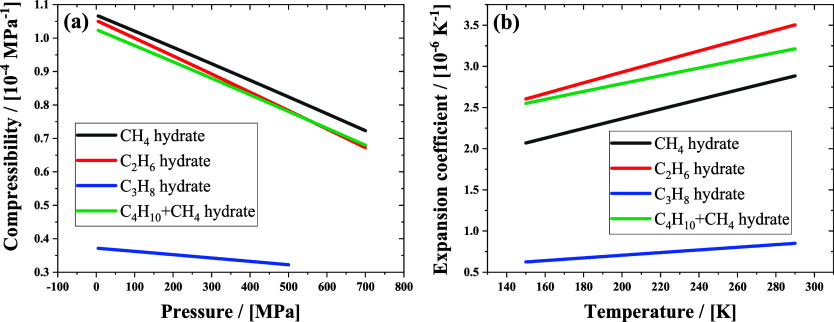
Isothermal compressibilities (a) and thermal expansion coefficients (b) of the hydrate crystals with the four *n*-alkanes in H-bonded cages.

By calculating the lattice parameters (shown in Figure 11), the thermal expansion coefficient (*α*_*P*_) and isothermal compressibility (*κ*_*T*_) of hydrates can be calculated through numerical differentiation using the following equations
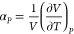
10
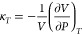
11where *V* is the volume of hydrate. The isothermal compressibilities and the thermal expansion coefficients of hydrates are shown in Figures 12 (a) and 12(b), respectively. The thermodynamic properties vary among hydrates formed with four different *n*-alkanes. For the four hydrates, propane hydrate has the lowest compressibility, because propane molecules only occupy the large cages in the sII hydrate crystal, leaving the small cage empty; there is no guest–host interaction in these small cages. The other three hydrates are all occupied, and the compressibilities of the hydrates are almost comparable to each other and much larger than that of propane hydrate (Figure 12a). For the expansion coefficient of hydrates as shown in Figure 12(b), ethane hydrate has the highest value of *α*_*P*_, followed by butane+methane binary hydrate, methane hydrate, and propane hydrate. Apart from propane hydrate, the other hydrates have similar thermal expansivity and compressibility. These results are consistent with Hester’s experiment results.^52^ Among these hydrates, only propane hydrate has empty small cages, as it is not fully occupied by guest molecules. This leads to the hydrate crystal being resistant to compression and expansion. The two thermal properties become less dependent on the guest size and hydrate crystal type and are instead dominated by guest occupancy.

## 4 Conclusions

The solubilities and diffusion coefficients of four light *n*-alkanes (methane, ethane, propane, and *n*-butane) in aqueous NaCl solutions and the thermodynamics properties of their corresponding hydrate crystals are calculated by molecular simulations. To improve accuracy of the calculations, CFCMC simulations were performed to correct the interaction between alkane groups and water by fitting the calculated and experimental values of the excess chemical potential of ethane and propane in water at 1 bar and 298.15 K. The optimized correction factor *k*_*ij*_ for the interactions between CH_3_ and water is 1.04, and *k*_*ij*_ between CH_2_ and water is 1.0. Using these correction factors, the excess chemical potentials of four light *n*-alkanes (methane, ethane, propane, and *n*-butane) in aqueous NaCl solutions with different molalities (0–6 mol/kg) and temperatures (278.15 K–308.15 K) are calculated at 1 bar, and the solubilities of the four alkanes in aqueous NaCl solution are calculated using Henry’s law. The solubilities of the four alkanes are calculated at high pressure (100, 200, and 300 bar) by using CFCMC simulations in the Gibbs Ensemble. All simulations are performed using the Brick-CFCMC software. Solubilities of the four alkanes depend on the temperature, pressure, and molality of NaCl in solution. Our simulation results show that the solubility of alkanes decreases with increasing salt molality, a phenomenon known as the salting out effect. The solubility data for each *n*-alkane at different conditions are listed in Table 3.

**Table 3:** Calculated Solubilities of the Four Light Alkanes in NaCl Solutions as a Function of NaCl Molality, Temperature, and Pressure^a^

			Methane		Ethane		Propane		Butane	
*T*	*P*	*M*	*S*	*u*(*s*)	*S*	*u*(*s***)**	*S*	*u*(*s*)	*S*	*u*(*s*)
278.15	1	0	**0.038**	**0.006**	**0.111**	**0.006**	**0.117**	**0.008**	**0.149**	**0.007**
278.15	1	0.6	**0.032**	**0.006**	**0.080**	**0.005**	**0.079**	**0.005**	**0.129**	**0.008**
278.15	1	1	**0.025**	**0.003**	**0.072**	**0.004**	**0.094**	**0.002**	**0.078**	**0.005**
278.15	1	2	**0.014**	**0.003**	**0.045**	**0.003**	**0.047**	**0.002**	**0.047**	**0.004**
278.15	1	4	**0.008**	**0.003**	**0.0155**	**0.0002**	**0.015**	**0.004**	**0.027**	**0.001**
278.15	1	6	**0.005**	**0.002**	**0.0067**	**0.0007**	**0.007**	**0.002**	**0.014**	**0.001**
288.15	1	0	**0.031**	**0.002**	**0.077**	**0.007**	**0.092**	**0.004**	**0.11**	**0.01**
288.15	1	0.6	**0.023**	**0.004**	**0.057**	**0.005**	**0.073**	**0.004**	**0.071**	**0.004**
288.15	1	1	**0.019**	**0.003**	**0.041**	**0.002**	**0.047**	**0.001**	**0.074**	**0.003**
288.15	1	2	**0.016**	**0.002**	**0.027**	**0.002**	**0.033**	**0.001**	**0.028**	**0.001**
288.15	1	4	**0.006**	**0.001**	**0.0110**	**0.0009**	**0.0118**	**0.005**	**0.021**	**0.002**
288.15	1	6	**0.0025**	**0.0005**	**0.0059**	**0.0006**	**0.0041**	**0.0002**	**0.0060**	**0.0005**
298.15	1	0	**0.023**	**0.003**	**0.0613**	**0.0008**	**0.070**	**0.002**	**0.064**	**0.005**
298.15	1	0.6	**0.021**	**0.003**	**0.0471**	**0.0009**	**0.059**	**0.002**	**0.0430**	**0.0006**
298.15	1	1	**0.018**	**0.002**	**0.039**_**1**_	**0.001**	**0.037**	**0.001**	**0.037**	**0.001**
298.15	1	2	**0.014**	**0.002**	**0.0243**	**0.0005**	**0.026**	**0.001**	**0.024**	**0.001**
298.15	1	4	**0.007**	**0.002**	**0.0106**	**0.0003**	**0.0101**	**0.0005**	**0.011**	**0.001**
298.15	1	6	**0.0033**	**0.0003**	**0.0057**	**0.0004**	**0.0045**	**0.0003**	**0.0039**	**0.0002**
308.15	1	0	**0.022**	**0.002**	**0.0489**	**0.0009**	**0.0475**	**0.0001**	**0.034**	**0.001**
308.15	1	0.6	**0.016**	**0.002**	**0.034**	**0.001**	**0.03882**	**0.00006**	**0.035**	**0.002**
308.15	1	1	**0.015**	**0.001**	**0.0341**	**0.0009**	**0.02857**	**0.00003**	**0.0267**	**0.0009**
308.15	1	2	**0.0111**	**0.0007**	**0.0209**	**0.0009**	**0.01818**	**0.00004**	**0.0156**	**0.0003**
308.15	1	4	**0.0061**	**0.0008**	**0.0087**	**0.0003**	**0.00669**	**0.00008**	**0.0060**	**0.0007**
308.15	1	6	**0.0030**	**0.0003**	**0.0042**	**0.0002**	**0.00371**	**0.00001**	**0.0018**	**0.0008**
278.15	100	0	**3.1**	**0.6**	**2.4**	**0.3**	**0.6**	**0.1**	**0.25**	**0.06**
278.15	100	0.6	**2.3**	**0.1**	**2.1**	**0.6**	**0.45**	**0.08**	**0.16**	**0.09**
278.15	100	1	**1.5**	**0.3**	**1.5**	**0.5**	**0.4**	**0.1**	**0.15**	**0.05**
278.15	100	2	**1.6**	**0.5**	**1.0**	**0.4**	**0.20**	**0.04**	**0.13**	**0.08**
278.15	100	4	**0.7**	**0.3**	**0.5**	**0.2**	**0.08**	**0.06**	**0.003**	**0.003**
278.15	100	6	**0.3**	**0.1**	**0.2**	**0.1**	**0.02**	**0.01**	**0.01**	**0.01**
288.15	100	0	**1.9**	**0.5**	**2.3**	**0.1**	**0.6**	**0.1**	**0.24**	**0.06**
288.15	100	0.6	**1.9**	**0.1**	**1.7**	**0.3**	**0.4**	**0.1**	**0.13**	**0.09**
288.15	100	1	**1.5**	**0.2**	**1.1**	**0.3**	**0.3**	**0.1**	**0.11**	**0.03**
288.15	100	2	**1.2**	**0.1**	**0.8**	**0.2**	**0.29**	**0.08**	**0.07**	**0.08**
288.15	100	4	**0.7**	**0.2**	**0.34**	**0.7**	**0.12**	**0.07**	**0.04**	**0.03**
288.15	100	6	**0.38**	**0.06**	**0.22**	**0.09**	**0.04**	**0.02**	**0.04**	**0.03**
298.15	100	0	**1.7**	**0.3**	**1.6**	**0.3**	**0.5**	**0.1**	**0.2**	**0.1**
298.15	100	0.6	**1.6**	**0.3**	**1.3**	**0.5**	**0.42**	**0.06**	**0.10**	**0.2**
298.15	100	1	**1.6**	**0.2**	**1.2**	**0.3**	**0.26**	**0.06**	**0.10**	**0.5**
298.15	100	2	**1.1**	**0.1**	**0.6**	**0.1**	**0.22**	**0.01**	**0.033**	**0.009**
298.15	100	4	**0.40**	**0.01**	**0.3**	**0.1**	**0.09**	**0.04**	**0.014**	**0.006**
298.15	100	6	**0.3**	**0.1**	**0.22**	**0.04**	**0.07**	**0.08**	**0.009**	**0.006**
308.15	100	0	**1.5**	**0.2**	**1.6**	**0.2**	**0.45**	**0.07**	**0.14**	**0.03**
308.15	100	0.6	**1.31**	**0.09**	**1.1**	**0.3**	**0.36**	**0.04**	**0.09**	**0.02**
308.15	100	1	**1.1**	**0.1**	**1.3**	**0.3**	**0.27**	**0.06**	**0.07**	**0.01**
308.15	100	2	**0.8**	**0.1**	**0.9**	**0.3**	**0.16**	**0.04**	**0.05**	**0.01**
308.15	100	4	**0.5**	**0.1**	**0.36**	**0.05**	**0.09**	**0.01**	**0.022**	**0.005**
308.15	100	6	**0.3**	**0.1**	**0.25**	**0.07**	**0.04**	**0.01**	**0.008**	**0.006**
278.15	200	0	**4.0**	**0.7**	**2.6**	**0.5**	**0.9**	**0.2**	**0.20**	**0.07**
278.15	200	0.6	**3.6**	**0.4**	**1.6**	**0.3**	**0.5**	**0.1**	**0.17**	**0.009**
278.15	200	1	**2.3**	**0.5**	**1.4**	**0.4**	**0.5**	**0.1**	**0.16**	**0.03**
278.15	200	2	**2.1**	**0.4**	**1.0**	**0.2**	**0.3**	**0.1**	**0.08**	**0.02**
278.15	200	4	**1.1**	**0.5**	**0.6**	**0.1**	**0.09**	**0.02**	**0.04**	**0.02**
278.15	200	6	**0.44**	**0.09**	**0.2**	**0.1**	**0.06**	**0.06**	**0.015**	**0.007**
288.15	200	0	**3.0**	**0.5**	**2.5**	**0.4**	**0.7**	**0.1**	**0.18**	**0.06**
288.15	200	0.6	**2.5**	**0.4**	**1.5**	**0.2**	**0.4**	**0.1**	**0.1**	**0.1**
288.15	200	1	**2.1**	**0.3**	**1.3**	**0.2**	**0.3**	**0.1**	**0.06**	**0.03**
288.15	200	2	**1.7**	**0.4**	**0.9**	**0.2**	**0.23**	**0.08**	**0.05**	**0.01**
288.15	200	4	**0.9**	**0.2**	**0.4**	**0.1**	**0.09**	**0.05**	**0.02**	**0.01**
288.15	200	6	**0.6**	**0.1**	**0.14**	**0.07**	**0.02**	**0.01**	**0.02**	**0.01**
298.15	200	0	**3.3**	**0.2**	**1.8**	**0.1**	**0.7**	**0.1**	**0.17**	**0.06**
298.15	200	0.6	**2.2**	**0.3**	**1.3**	**0.1**	**0.45**	**0.06**	**0.14**	**0.02**
298.15	200	1	**1.9**	**0.4**	**1.3**	**0.4**	**0.25**	**0.06**	**0.10**	**0.05**
298.15	200	2	**1.4**	**0.4**	**0.7**	**0.2**	**0.27**	**0.07**	**0.05**	**0.03**
298.15	200	4	**1.0**	**0.4**	**0.40**	**0.03**	**0.11**	**0.02**	**0.023**	**0.008**
298.15	200	6	**0.37**	**0.07**	**0.22**	**0.08**	**0.05**	**0.03**	**0.009**	**0.002**
308.15	200	0	**2.6**	**0.3**	**1.9**	**0.1**	**0.7**	**0.1**	**0.14**	**0.03**
308.15	200	0.6	**2.2**	**0.5**	**1.5**	**0.1**	**0.48**	**0.09**	**0.11**	**0.02**
308.15	200	1	**1.9**	**0.4**	**1.0**	**0.2**	**0.4**	**0.2**	**0.10**	**0.01**
308.15	200	2	**1.7**	**0.3**	**0.8**	**0.2**	**0.19**	**0.09**	**0.07**	**0.02**
308.15	200	4	**0.8**	**0.1**	**0.37**	**0.09**	**0.11**	**0.04**	**0.02**	**0.01**
308.15	200	6	**0.4**	**0.1**	**0.17**	**0.08**	**0.04**	**0.02**	**0.014**	**0.007**
278.15	300	0	**4.9**	**0.6**	**2.8**	**0.5**	**0.8**	**0.1**	**0.35**	**0.04**
278.15	300	0.6	**3.8**	**0.2**	**1.8**	**0.5**	**0.7**	**0.1**	**0.20**	**0.07**
278.15	300	1	**2.9**	**0.4**	**1.7**	**0.2**	**0.64**	**0.09**	**0.15**	**0.04**
278.15	300	2	**2.2**	**0.3**	**1.3**	**0.2**	**0.3**	**0.1**	**0.10**	**0.03**
278.15	300	4	**1.1**	**0.1**	**0.7**	**0.1**	**0.12**	**0.06**	**0.08**	**0.05**
278.15	300	6	**0.5**	**0.1**	**0.20**	**0.07**	**0.07**	**0.04**	**0.01**	**0.01**
288.15	300	0	**3.9**	**0.5**	**2.7**	**0.6**	**0.9**	**0.2**	**0.25**	**0.03**
288.15	300	0.6	**3.3**	**0.5**	**2.1**	**0.1**	**0.6**	**0.1**	**0.18**	**0.03**
288.15	300	1	**2.8**	**0.4**	**1.6**	**0.6**	**0.3**	**0.1**	**0.13**	**0.05**
288.15	300	2	**2.3**	**0.3**	**1.3**	**0.7**	**0.41**	**0.06**	**0.10**	**0.06**
288.15	300	4	**1.3**	**0.6**	**0.8**	**0.5**	**0.11**	**0.07**	**0.02**	**0.01**
288.15	300	6	**0.6**	**0.5**	**0.2**	**0.1**	**0.06**	**0.02**	**0.01**	**0.01**
298.15	300	0	**3.7**	**0.3**	**2.2**	**0.2**	**0.60**	**0.05**	**0.19**	**0.05**
298.15	300	0.6	**2.9**	**0.3**	**1.6**	**0.2**	**0.47**	**0.06**	**0.15**	**0.04**
298.15	300	1	**2.5**	**0.5**	**1.65**	**0.08**	**0.26**	**0.02**	**0.13**	**0.04**
298.15	300	2	**1.7**	**0.3**	**0.8**	**0.1**	**0.25**	**0.03**	**0.07**	**0.02**
298.15	300	4	**0.9**	**0.2**	**0.4**	**0.2**	**0.12**	**0.03**	**0.03**	**0.02**
298.15	300	6	**0.5**	**0.1**	**0.19**	**0.07**	**0.04**	**0.02**	**0.02**	**0.02**
308.15	300	0	**2.8**	**0.6**	**1.9**	**0.3**	**0.66**	**0.06**	**0.16**	**0.04**
308.15	300	0.6	**2.6**	**0.6**	**1.5**	**0.2**	**0.47**	**0.05**	**0.097**	**0.008**
308.15	300	1	**2.3**	**0.3**	**1.3**	**0.3**	**0.3**	**0.1**	**0.104**	**0.007**
308.15	300	2	**1.7**	**0.3**	**0.9**	**0.2**	**0.20**	**0.07**	**0.06**	**0.02**
308.15	300	4	**1.1**	**0.2**	**0.5**	**0.2**	**0.14**	**0.06**	**0.02**	**0.01**
308.15	300	6	**0.4**	**0.1**	**0.3**	**0.1**	**0.04**	**0.01**	**0.013**	**0.005**

a *T* is temperature in units of K; *P* is the pressure in units of bar; *M* is the molality of NaCl in units of mol/kg; *S* is solubility; *u*(*s*) is the standard uncertainty, and the units of *S* and *u*(*s*) are g/kg. These uncertainties are calculated based on the results of five independent simulations for each condition (concentration, temperature, and pressure).

Using the correction factors *k*_*ij*_, MD simulations are performed to calculate the transport properties of the four light *n*-alkanes in aqueous NaCl solutions at 1 bar and temperatures of 278.15 K–308.15 K. The shear viscosities of the aqueous solution systems with the alkanes and diffusion coefficients (*D*_Self_) optimized by a finite-size correction of the four alkanes in solution with different NaCl molalities are obtained. The diffusion coefficients decrease as the carbon number increases in *n*-alkanes and as the system temperature decreases. The viscosity and diffusion coefficient data for each *n*-alkane at different conditions can be found in Table 4.

**Table 4:** Calculated Viscosities of Aqueous NaCl Solutions with One Alkane Molecule in Systems and Self-Diffusion Coefficients for the Four Alkanes in NaCl Solutions as a Function of Temperature and Pressure^a^

			Methane		Ethane		Propane		Butane	
*T*	*P*	*M*	η	*u*(η)	η	*u*(η)	η	*u*(η)	η	*u*(η)
278.15	1	0	**1.42**	**0.04**	**1.42**	**0.05**	**1.41**	**0.07**	**1.6**	**0.2**
278.15	1	0.6	**1.51**	**0.04**	**1.64**	**0.08**	**1.58**	**0.08**	**1.5**	**0.1**
278.15	1	1	**1.68**	**0.04**	**1.73**	**0.05**	**1.60**	**0.03**	**1.8**	**0.1**
278.15	1	2	**2.02**	**0.05**	**2.0**	**0.1**	**2.1**	**0.2**	**2.2**	**0.1**
278.15	1	4	**2.8**	**0.3**	**3.0**	**0.3**	**3.0**	**0.4**	**3.3**	**0.4**
278.15	1	6	**5.2**	**0.9**	**5.1**	**0.9**	**5.2**	**0.3**	**5.5**	**0.5**
288.15	1	0	**1.10**	**0.07**	**1.1**	**0.1**	**1.05**	**0.03**	**1.10**	**0.06**
288.15	1	0.6	**1.27**	**0.09**	**1.2**	**0.1**	**1.20**	**0.01**	**1.27**	**0.09**
288.15	1	1	**1.4**	**0.1**	**1.21**	**0.03**	**1.27**	**0.03**	**1.38**	**0.09**
288.15	1	2	**1.51**	**0.03**	**1.53**	**0.03**	**1.26**	**0.07**	**1.4**	**0.1**
288.15	1	4	**2.43**	**0.04**	**2.2**	**0.1**	**2.17**	**0.06**	**2.3**	**0.2**
288.15	1	6	**3.43**	**0.03**	**3.2**	**0.1**	**3.6**	**0.4**	**3.6**	**0.4**
298.15	1	0	**0.85**	**0.02**	**0.83**	**0.03**	**0.86**	**0.05**	**0.88**	**0.04**
298.15	1	0.6	**1.03**	**0.02**	**0.98**	**0.06**	**0.86**	**0.08**	**1.03**	**0.06**
298.15	1	1	**1.05**	**0.05**	**1.00**	**0.06**	**1.1**	**0.1**	**1.07**	**0.08**
298.15	1	2	**1.3**	**0.1**	**1.21**	**0.03**	**1.22**	**0.07**	**1.19**	**0.01**
298.15	1	4	**1.66**	**0.05**	**1.72**	**0.05**	**1.8**	**0.1**	**1.6**	**0.1**
298.15	1	6	**2.3**	**0.1**	**2.43**	**0.06**	**2.5**	**0.1**	**2.4**	**0.3**
308.15	1	0	**0.783**	**0.002**	**0.69**	**0.03**	**0.72**	**0.05**	**0.74**	**0.09**
308.15	1	0.6	**0.7**	**0.1**	**0.78**	**0.05**	**0.76**	**0.02**	**0.7**	**0.1**
308.15	1	1	**0.79**	**0.01**	**0.80**	**0.05**	**0.9**	**0.1**	**0.83**	**0.02**
308.15	1	2	**0.93**	**0.04**	**0.99**	**0.07**	**0.99**	**0.07**	**1.02**	**0.06**
308.15	1	4	**1.33**	**0.05**	**1.35**	**0.02**	**1.36**	**0.04**	**1.32**	**0.05**
308.15	1	6	**1.8**	**0.1**	**1.87**	**0.04**	**1.9**	**0.1**	**1.79**	**0.08**

			Methane		Ethane		Propane		Butane	
*T*	*P*	*M*	*D*_Self_	*u*(*D*_Self_)	*D*_Self_	*u*(*D*_Self_)	*D*_Self_	*u*(*D*_Self_)	*D*_Self_	*u*(*D*_Self_)
278.15	1	0	**1.1**	**0.1**	**0.8**	**0.2**	**0.78**	**0.04**	**0.6**	**0.1**
278.15	1	0.6	**0.96**	**0.04**	**0.75**	**0.04**	**0.81**	**0.05**	**0.60**	**0.03**
278.15	1	1	**0.88**	**0.03**	**0.7**	**0.1**	**0.7**	**0.1**	**0.50**	**0.09**
278.15	1	2	**0.87**	**0.06**	**0.56**	**0.04**	**0.5**	**0.2**	**0.37**	**0.06**
278.15	1	4	**0.4**	**0.1**	**0.47**	**0.01**	**0.41**	**0.06**	**0.37**	**0.06**
278.15	1	6	**0.33**	**0.04**	**0.35**	**0.03**	**0.15**	**0.06**	**0.15**	**0.07**
288.15	1	0	**1.4**	**0.1**	**1.07**	**0.06**	**1.1**	**0.1**	**0.97**	**0.06**
288.15	1	0.6	**1.3**	**0.1**	**1.0**	**0.1**	**1.1**	**0.2**	**0.83**	**0.04**
288.15	1	1	**0.9**	**0.2**	**1.01**	**0.03**	**1.0**	**0.1**	**0.7**	**0.1**
288.15	1	2	**0.9**	**0.1**	**0.7**	**0.2**	**1.03**	**0.06**	**0.6**	**0.1**
288.15	1	4	**0.7**	**0.1**	**0.7**	**0.1**	**0.6**	**0.1**	**0.4**	**0.1**
288.15	1	6	**0.5**	**0.1**	**0.49**	**0.06**	**0.46**	**0.04**	**0.31**	**0.09**
298.15	1	0	**1.97**	**0.06**	**1.5**	**0.2**	**1.2**	**0.2**	**1.19**	**0.05**
298.15	1	0.6	**1.7**	**0.1**	**1.3**	**0.3**	**1.31**	**0.03**	**1.1**	**0.1**
298.15	1	1	**1.6**	**0.2**	**1.3**	**0.1**	**1.26**	**0.07**	**1.1**	**0.2**
298.15	1	2	**1.5**	**0.1**	**1.1**	**0.2**	**1.2**	**0.2**	**0.9**	**0.1**
298.15	1	4	**1.0**	**0.1**	**0.8**	**0.1**	**0.8**	**0.2**	**0.8**	**0.2**
298.15	1	6	**0.82**	**0.02**	**0.7**	**0.1**	**0.5**	**0.1**	**0.59**	**0.04**
308.15	1	0	**2.4**	**0.1**	**1.9**	**0.1**	**1.6**	**0.3**	**1.67**	**0.07**
308.15	1	0.6	**2.2**	**0.1**	**1.9**	**0.1**	**1.5**	**0.3**	**1.5**	**0.1**
308.15	1	1	**2.0**	**0.1**	**1.6**	**0.2**	**1.5**	**0.2**	**1.4**	**0.5**
308.15	1	2	**1.9**	**0.1**	**1.48**	**0.03**	**1.4**	**0.2**	**1.2**	**0.2**
308.15	1	4	**1.4**	**0.2**	**1.2**	**0.2**	**1.0**	**0.2**	**0.8**	**0.3**
308.15	1	6	**0.8**	**0.3**	**0.9**	**0.1**	**0.8**	**0.1**	**0.6**	**0.1**

a *T* is temperature in units of K; *P* is the pressure in units of bar; *M* is the molality of NaCl in units of mol/kg; η is shear viscosity; *u*(η) is the standard uncertainty; the units of η and *u*(η) are mPa·s; *D*_Self_ is the self-diffusion coefficient; *u*(*D*_Self_) is the standard uncertainty; and the units of *D*_Self_ and *u*(*D*_Self_) are 10^–9^ m^2^/s. These uncertainties are calculated based on the results of five independent simulations for each condition (concentration, temperature, and pressure).

The lattice parameters of the four corresponding hydrates (methane hydrate, ethane hydrate, propane hydrate, and butane+methane binary hydrate) are computed for a wide range of temperatures (150–300) K and pressures (1–7000 bar) using MD simulations. The thermodynamic properties of the hydrates, including isothermal compressibility and the thermal expansion coefficient, are calculated by the numerical differentiation of the hydrate volume. The thermodynamic properties of the four hydrates differ from each other, particularly in the case of the propane hydrate due to the effects of host–guest interactions and guest occupancy.

## Supporting Information Available

Parameters for the force fields; Fugacity coefficients for the four light alkanes; excess chemical potentials of methane, ethane, and propane in water; solubilities of ethane, propane, and butane in the NaCl solutions; viscosities of NaCl solutions and self-diffusion data of methane at pressures 1–300 bar (PDF)
