# Supplementary material for: Solubilities and Self-Diffusion Coefficients of Light n-Alkanes in NaCl Solutions at the Temperature Range (278.15–308.15) K and Pressure Range (1–300) bar and Thermodynamics Properties of Their Corresponding Hydrates at (150–290) K and (1–7000) bar
Source: J Chem Eng Data. 2023 Jul 11;69(10):3330–46. doi: 10.1021/acs.jced.3c00225 (PMC11472311; doi:10.1021/acs.jced.3c00225)
Supplement: Supplementary file 1 — je3c00225_si_001.pdf [file je3c00225_si_001.pdf]

Supporting Information for:

Solubilities and Self-diffusion Coefficients of Light n-alkanes in NaCl Solutions at the temperature range (278.15 − 308.15) K and pressure range (1 − 300) bar and Thermodynamics Properties of Their Corresponding Hydrates at (150 − 290) K and (1 − 7000) bar

Bin Fang[a,b], Parsa Habibi[b], Othonas A. Moultos[b]*, Tao Lü[c,d], Fulong Ning[e,f,g], Thijs J.H. Vlugt[b]**

[a] School of Mathematics and Physics, China University of Geosciences, Wuhan 430074, China

[b] Engineering Thermodynamics, Process & Energy Department, Faculty of Mechanical, Maritime and Materials Engineering, Delft University of Technology, Leeghwaterstraat 39, Delft 2628CB, The Netherlands

[c] School of Automation, China University of Geosciences, Wuhan 430074, China

[d] Hubei Key Laboratory of Advanced Control and Intelligent Automation for Complex Systems, Wuhan 430074, China

[e] Faculty of Engineering, China University of Geosciences, Wuhan, Hubei 430074, China

[f] National Center for International Research on Deep Earth Drilling and Resource Development, China University of Geosciences, Wuhan 430074, China

[g] Laboratory for Marine Mineral Resources, Qingdao National Laboratory for Marine Science and Technology, Qingdao 266237, China

*Corresponding author: O.Moultos@tudelft.nl (Othonas A. Moultos)

**Corresponding author: T.J.H.Vlugt@tudelft.nl (Thijs J.H. Vlugt)

Table S1. Non-bonded parameters for the TIP4P/2005[1] water force field, methane-methane, and methane-water interactions optimized by Docherty. [2] σ and ε are the Lennard-Jones parameters, in unit of Å and K, respectively; q is the partial charge of an atom in units of elementary charge (e); r is the distance between particles in units of Å

**Model**	ε/k_B **(K)**	σ **(Å)**	q_H **(e)**	r_{OM} **(Å)**	r_{OH} **(Å)**	**∠HOH,** deg
**Water TIP4P/2005**	93.2	3.1589	0.5564	0.1546	0.9572	104.52
**CH4**	147.5	3.73				
**Water-CH4 (k_{ij}=1.07)**	125.45	3.4445				

Table S2. Non-bonded parameters for the TraPPE[3] alkane force field, and Madrid-2019 ion forcefield.[4] σ and ε are the Lennard-Jones parameters, in unit of Å and K, respectively; q is the partial charge of an atom in units of elementary charge (e).

**Pseudo (atom)**	ε/k_B **(K)**	σ **(Å)**	q_H **(e)**	**Force field**
**CH3**	98	3.75		TraPPE
**CH2**	46	3.95		
**Na^+**	177.1257	2.21737	0.85	Madrid-2019
**Cl^-**	9.256	4.69906	-0.85	
**Na^+– Cl^-**	173.142	3.00512		
**Na^+–water**	95.4683	2.60838		
**Cl^-–water**	7.4584	4.23867		

Table S3. Bonded parameters for the TraPPE[3] alkane force field. Fixed bond lengths are used; The bond-bending energy is calculated according to: $U_{bend}(\theta) = \frac{k_\theta}{2}(\theta - \theta_{eq})^2$, where k_θ is the force constant, θ and θ_{eq} are the angle and the equilibrium angle, respectively; The torsion energy is calculated as: $U_{torsion}(\phi) = c_0 + c_1[1+\cos(\phi)] + c_2[1-\cos(2\phi)] + c_3[1+\cos(3\phi)]$, where c_0, c_1, c_2, c_3 are constants; ϕ represents the dihedral angle, which corresponds to synperiplanar (*cis*) conformations when ϕ=0°, and antiperiplanar (*trans*) conformations when ϕ=180°.

**Bond-stretching interaction**	
Type	***r* (Å)**
**CH3-CH3**	1.54
**CH3-CH2**	1.54

**Bond-bending interaction**			
Type	θ_{eq} (deg)	k_θ/k_B (K/rad^2)	
**CH3-CH2-CH3**	114	62500	
**CH3-CH2-CH2**	114	62500	

**Dihedral torsion parameter**				
Type	c_0/k_B (K)	c_1/k_B (K)	c_2/k_B (K)	c_3/k_B (K)
CH3-CH2-CH2-CH3	0.00	355.03	-68.19	791.32

Table S4. Fugacity coefficients obtained by REFPROP[5] for the four light alkanes at different temperatures and pressures

P (bar) / T (K)	100 bar	phase	200 bar	phase	300 bar	phase
	**Methane**					
278.15	0.80660	G	0.68713	G	0.63856	G
288.15	0.82922	G	0.72169	G	0.67625	G
298.15	0.84912	G	0.75297	G	0.71128	G
308.15	0.86672	G	0.78123	G	0.74371	G
	**Ethane**					
278.15	0.25544	L	0.17176	L	0.15184	L
288.15	0.30216	L	0.20323	L	0.17925	L
298.15	0.35124	L	0.23667	L	0.20841	L
308.15	0.40173	G	0.27168	G	0.23904	G
	**Propane**					
278.15	0.069011	L	0.048906	L	0.045842	L
288.15	0.088635	L	0.062493	L	0.058231	L
298.15	0.11135	L	0.078173	L	0.07246	L
308.15	0.13715	L	0.095941	L	0.08852	L
	**n-butane**					
278.15	0.017921	L	0.0135	L	0.013472	L
288.15	0.024859	L	0.018576	L	0.01838	L
298.15	0.033547	L	0.024887	L	0.024434	L
308.15	0.04417	L	0.032556	L	0.031738	L

P is pressure; T is temperature; G represents gas phase; L represent liquid phase.

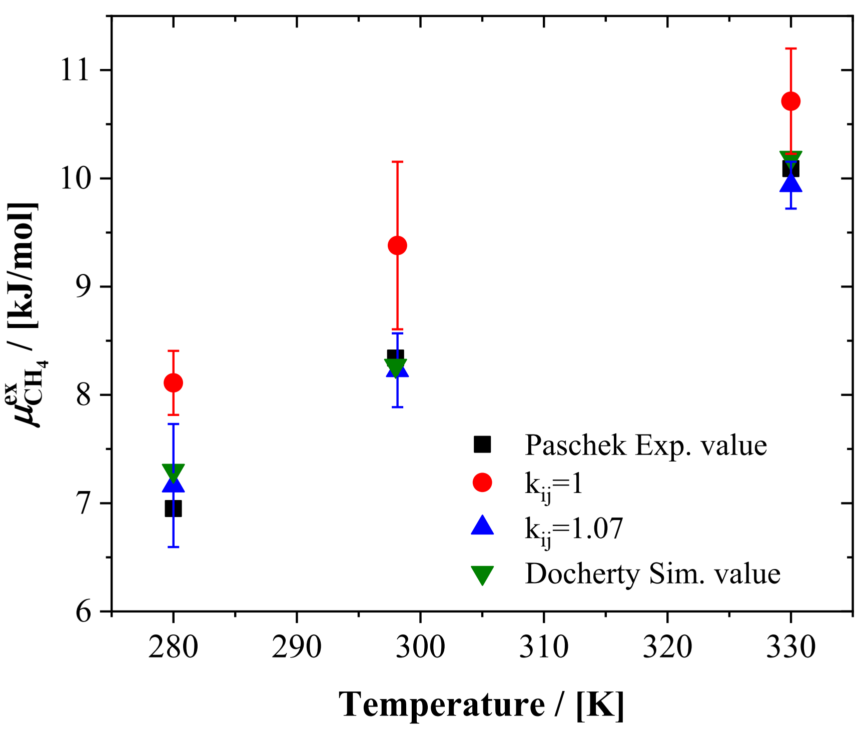

Figure S1. Excess chemical potential of CH_4 in water at 1 bar as a function of temperature. Docherty's simulation data and Paschek's experimental data are obtained from Ref. [2][2] and Ref. [6][6] respectively.

Table S5. Calculated excess chemical potential μ_{ex} for ethane in water at 298.15 K and 1 bar using different force field combinations. The reference value from experimental data is equal to 7.59 kJ/mol[7]

Water model	C_2H_6 model	simulation value	difference
**TIP4P2005[1]**	**TraPPE[3]**	**8.38±0.49**	**0.79**
**TIP4P2005[1]**	**HH-alkane[8]**	**7.04±0.067**	**-0.55**
**TIP4P2005[1]**	**NERE[9]**	**9.02±0.2**	**1.43**
**SPC/E[10]**	**TraPPE[3]**	**8.51±0.40**	**0.92**
**SPC/E[10]**	**NERE[9]**	**8.42±0.28**	**0.83**
**SPC[11]**	**TraPPE[3]**	**8.21±0.28**	**0.62**
**SPC[11]**	**NERE-0.035[12]**	**19.45±0.08**	**11.86**
**SPC[11]**	**NERE-0.083[12]**	**7.14±0.04**	**-0.45**

Table S6. Calculated excess chemical potential μ_{ex} for propane in water at 298.15 K and 1 bar using different force fields for propane

Exp. value[7]	TraPPE[3]	HH-alkane[8]	NERE[9]
8.15	9.5±0.64	8.88±1.14	9.34

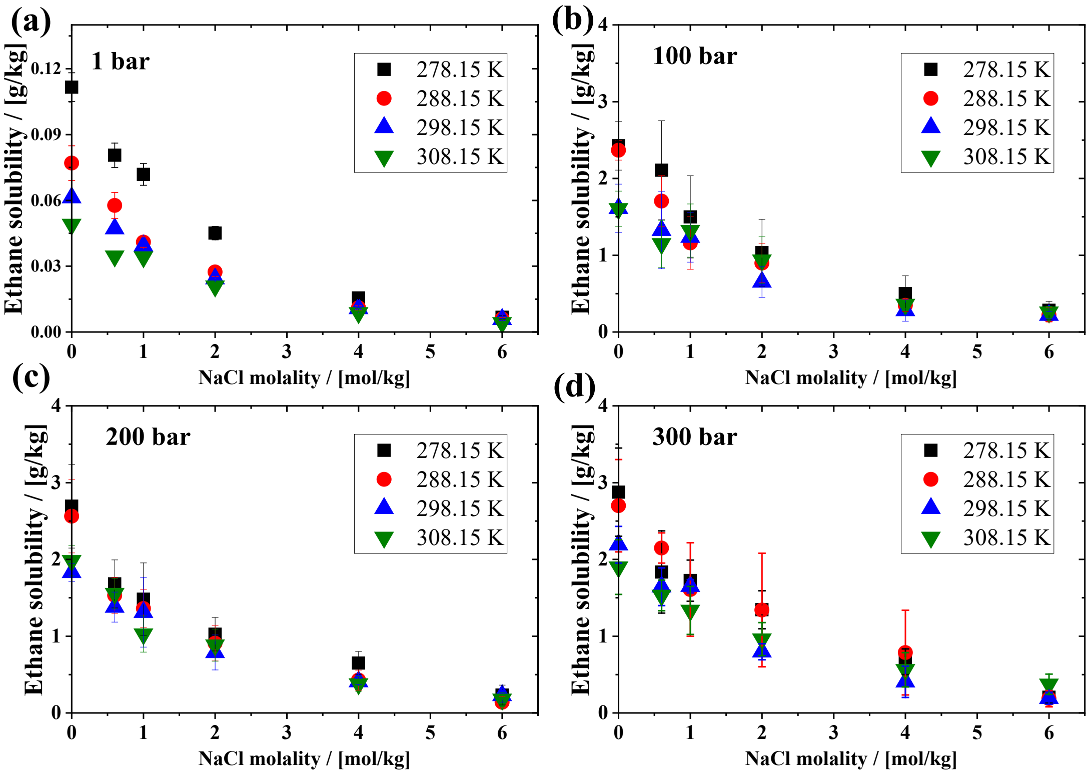

Figure S2. Solubilities of ethane in aqueous NaCl solutions as a function of the NaCl molality at different pressures ((a)1bar, (b)100 bar, (c) 200 bar, (d) 300bar) and temperatures (278.15-308.15 K).

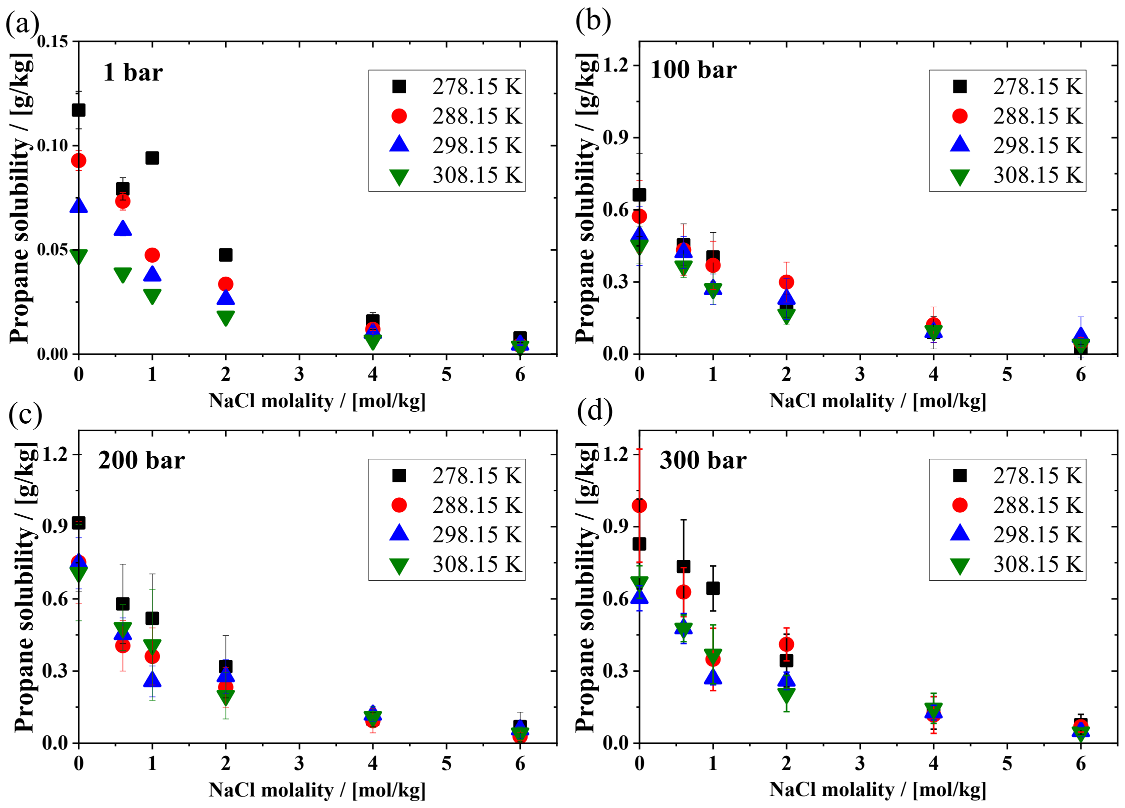

Figure S3. Solubilities of propane in aqueous NaCl solutions as a function of the NaCl molality at different pressures ((a)1bar, (b)100 bar, (c) 200 bar, (d) 300bar) and temperatures (278.15-308.15 K).

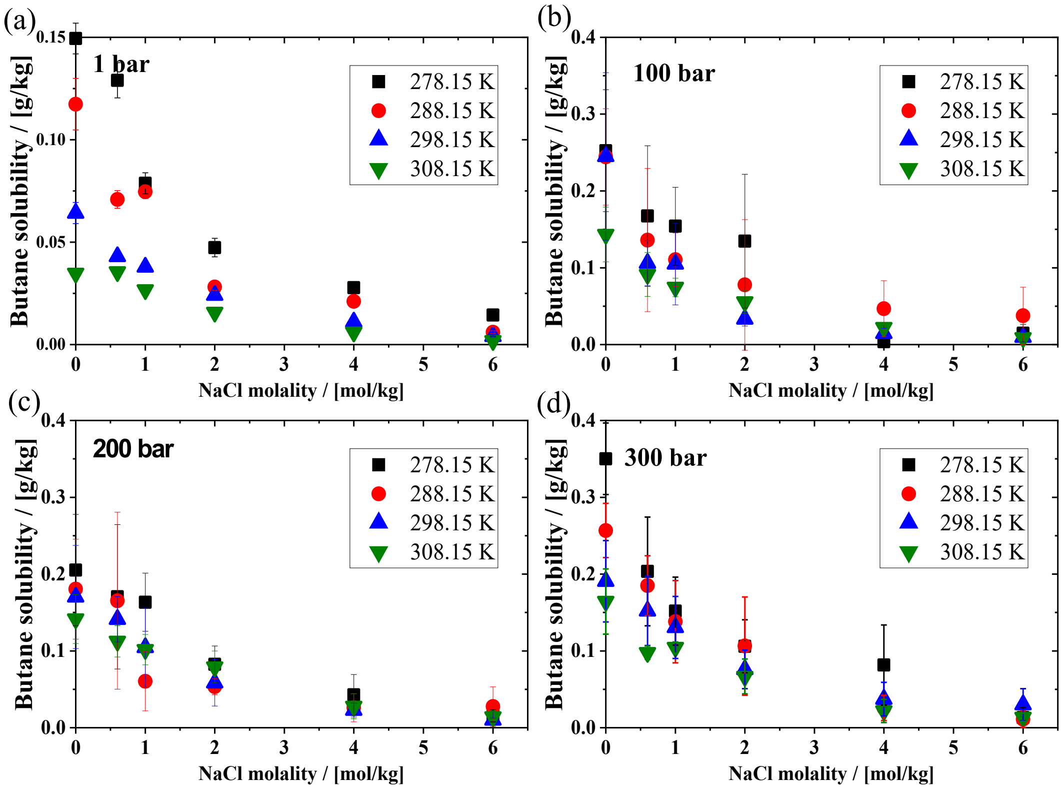

Figure S4. Solubilities of butane in aqueous NaCl solutions as a function of the NaCl molality at different pressures ((a)1bar, (b)100 bar, (c) 200 bar, (d) 300bar) and temperatures (278.15-308.15) K.

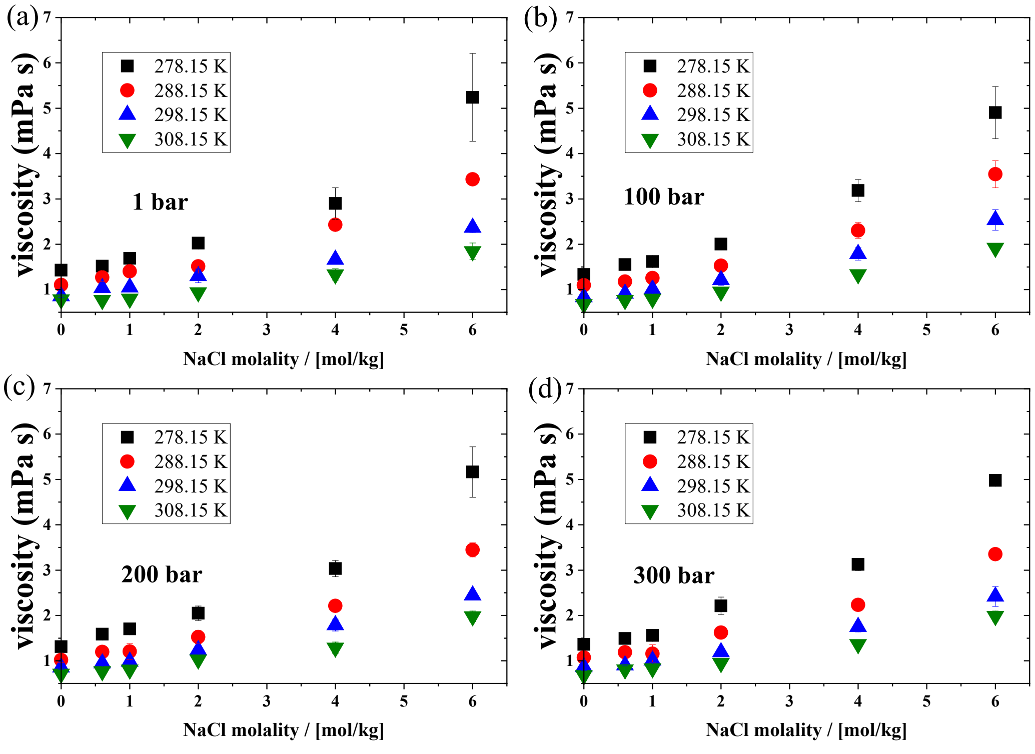

Figure S5. Calculated viscosities of aqueous NaCl solutions with one methane molecule at different temperatures and pressures ((a) 1 bar, (b) 100 bar, (c) 200 bar, (d) 300 bar) as a function of the NaCl molality.

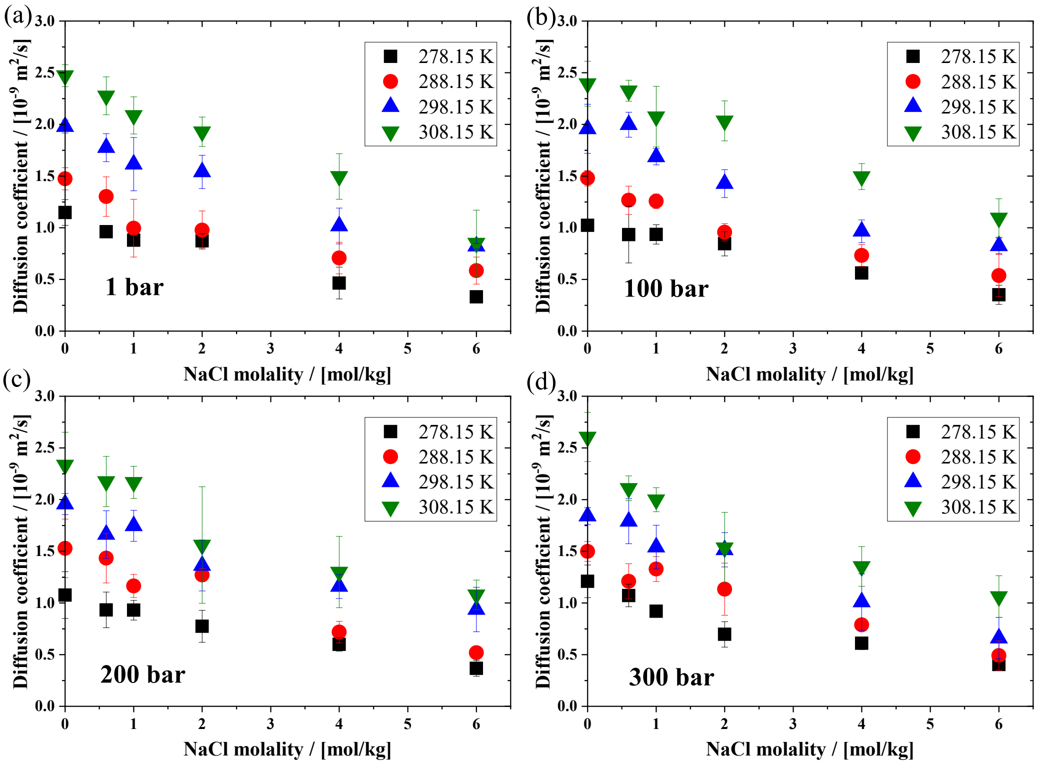

Figure S6. Calculated self-diffusion coefficients of methane in aqueous NaCl solutions at different temperatures and pressures ((a) 1 bar, (b) 100 bar, (c) 200 bar, (d) 300 bar) as a function of NaCl molality.

(1) Abascal, J. L. F.; Vega, C. A general purpose model for the condensed phases of water: TIP4P/2005. *J. Chem. Phys.* 2005, *123* (23), 234505

(2) Docherty, H.; Galindo, A.; Vega, C.; Sanz, E. A potential model for methane in water describing correctly the solubility of the gas and the properties of the methane hydrate. *J. Chem. Phys.* 2006, *125* (7), 074510

(3) Martin, M. G.; Siepmann, J. I. Transferable Potentials for Phase Equilibria. 1. United-Atom Description of n-Alkanes. *J. Phys. Chem. B* 1998, *102* (14), 2569-2577

(4) Zeron, I. M.; Abascal, J. L. F.; Vega, C. A force field of Li^+, Na^+, K^+, Mg^{2+}, Ca^{2+}, Cl^-, and SO_4^{2-} in aqueous solution based on the TIP4P/2005 water model and scaled charges for the ions. *J. Chem. Phys.* 2019, *151* (13), 134504

(5) Eric, L.; Marcia, H.; Mark, M. NIST Standard Reference Database 23: Reference Fluid Thermodynamic and Transport Properties-REFPROP, Version 9.1. Natl Std. Ref. Data Series (NIST NSRDS), National Institute of Standards and Technology, Gaithersburg, MD: 2013.

(6) Paschek, D. Temperature dependence of the hydrophobic hydration and interaction of simple solutes: An examination of five popular water models. *J. Chem. Phys.* 2004, *120* (14), 6674-6690

(7) Wilhelm, E.; Battino, R.; Wilcock, R. J. Low-pressure solubility of gases in liquid water. *Chem. Rev.* 1977, *77* (2), 219-262

(8) Ashbaugh, H. S.; Liu, L.; Surampudi, L. N. Optimization of linear and branched alkane interactions with water to simulate hydrophobic hydration. *J. Chem. Phys.* 2011, *135* (5), 054510

(9) Nath, S. K.; Escobedo, F. A.; de Pablo, J. J. On the simulation of vapor–liquid equilibria for alkanes. *J. Chem. Phys.* 1998, *108* (23), 9905-9911

(10) Berendsen, H. J. C.; Grigera, J. R.; Straatsma, T. P. The missing term in effective pair potentials. *J. Phys. Chem.* 1987, *91* (24), 6269-6271

(11) Berendsen, H. J. C.; Postma, J. P. M.; van Gunsteren, W. F.; Hermans, J. Interaction Models for Water in Relation to Protein Hydration. In *Intermolecular Forces: Proceedings of the Fourteenth Jerusalem Symposium on Quantum Chemistry and Biochemistry Held in Jerusalem, Israel, April 13–16, 1981*, Pullman, B. Ed.; Springer Netherlands, 1981; pp 331-342.

(12) Torres-García, G.; Luis, D. P.; Odriozola, G.; López-Lemus, J. Ethane clathrates using different water–ethane models: Molecular dynamics. *Physica A* 2018, *491*, 89-100
